# Residential Proximity Land Use Characteristics and Exhaled Volatile Organic Compounds’ Impact on Pulmonary Function in Asthmatic Children

**DOI:** 10.3390/jox15010027

**Published:** 2025-02-05

**Authors:** Bo-Yu Hsiao, Chun-Sheng Huang, Chang-Fu Wu, Kuo-Liong Chien, Hsiao-Yu Yang

**Affiliations:** 1 Population Health Research Center, National Taiwan University, Taipei 10055, Taiwan; d05849018@ntu.edu.tw (B.-Y.H.); changfu@ntu.edu.tw (C.-F.W.); klchien@ntu.edu.tw (K.-L.C.); 2 Institute of Epidemiology and Preventive Medicine, College of Public Health, National Taiwan University, Taipei 10055, Taiwan; 3 Institute of Environmental and Occupational Health Sciences, College of Public Health, National Taiwan University, Taipei 10055, Taiwan; d08852003@ntu.edu.tw; 4 Department of Public Health, College of Public Health, National Taiwan University, Taipei 10055, Taiwan; 5 Department of Environmental and Occupational Medicine, National Taiwan University Hospital, Taipei 100225, Taiwan; 6 Department of Community and Family Medicine, National Taiwan University Hospital Yunlin Branch, Yunlin 640, Taiwan

**Keywords:** exhaled volatile organic compounds, land use variables, pulmonary function, asthmatic children, biomarkers

## Abstract

Background: Urban air pollution adversely affects children’s respiratory systems, but the impact of volatile organic compounds (VOCs) on children’s pulmonary function remains unclear. This study aims to identify exhaled VOCs linked to land use characteristics and reduced pulmonary function in asthmatic children, as well as to explore environmental thresholds influencing VOC exposure levels. Methods: We enrolled 97 asthmatic children, aged 7 to 20, from Changhua County, Taiwan, and collected personal and residential data, collected exhaled VOC samples, and conducted pulmonary function tests. Land use characteristics were derived from the children’s residential addresses. This study used two models to explore the relationships between land use, VOC levels, and pulmonary function. Results: Our results show that m/p-xylene, 1,3,5-trimethylbenzene, and 1,2,4-trimethylbenzene were key contributors to FEV_1_/FVC and significantly predicted FEV_1_/FVC < 90% (AUC = 0.66; 95% CI: 0.53 to 0.79). These VOCs were also linked to major road areas within a 300 m buffer around children’s homes. Conclusions: This study fills a research gap on low-level outdoor VOC exposure and pediatric respiratory health, examining 1,3,5-trimethylbenzene, 1,2,4-trimethylbenzene, and m/p-xylene as potential biomarkers for impaired pulmonary function in children.

## 1. Introduction

Urbanization and industrial activities have raised significant concerns about air pollution’s impact on health, particularly its pronounced effects on the respiratory system [1,2]. Due to their higher ventilation rate per unit body weight and increased outdoor activity time, children are especially vulnerable to air pollution [3,4]. Therefore, safeguarding children’s respiratory health is of paramount importance. Many respiratory diseases, such as asthma and chronic obstructive pulmonary disease (COPD), often present with early signs of airway narrowing before the full onset of the disease [5,6]. This presents an opportunity for the early prevention of respiratory diseases in children; identifying key modifiable factors associated with airway narrowing could effectively prevent disease development.

Air pollution is composed of multiple constituents, including PM_2.5_, SO_2_, NO_x_, ozone, carbon monoxide, lead, volatile organic compounds (VOCs), and other substances. VOCs are particularly significant because they can act as precursors to PM and ozone [7,8]. VOCs can be detected in exhaled breath, which directly reflects the physiological or pathological conditions within the body [9,10]. Consequently, there is growing interest in utilizing exhaled VOCs as biomarkers for diagnosing respiratory diseases in clinical settings [11,12], with studies focusing on conditions such as asthma [13], COPD [14], lung cancer [15], and even acute infectious diseases like COVID-19 [16]. However, research exploring the use of exhaled VOCs as biomarkers for detecting pulmonary function abnormalities, particularly in children, remains limited [17].

Xenobiotics refer to any chemical substances that are foreign to a living organism, typically including compounds that are not naturally produced by the organism [18]. Due to their foreign nature, xenobiotics can potentially affect the health of organisms and ecosystems, making them significant in the fields of toxicology and environmental science. Air pollutants are xenobiotics because they are foreign chemical substances not naturally produced by living organisms, including VOCs. Detecting xenobiotics in environmental samples is challenging due to their low concentrations, which make them difficult to identify. Moreover, these airborne compounds can penetrate the respiratory system [19]. Xenobiotic metabolism of ambient air pollutants plays a key role in the development and exacerbation of asthma [20]. However, there is currently no method to measure the internal doses of inhaled air pollutants in the lungs, thus limiting the ability to explore their mechanisms and identify potential biomarkers for assessing xenobiotic health hazards. The external exposure to VOCs can be highly variable and influenced by numerous factors, including fluctuating and irregular sources that are challenging to detect with conventional environmental monitoring methods. This variability underscores the importance of exhaled breath analysis as a valuable tool for assessing internal exposure to xenobiotics. The unique advantage of exhaled breath analysis is its ability to capture internal exposure levels, particularly in complex and fluctuating environments. By directly reflecting the internal dose of VOCs, exhaled breath analysis provides a more reliable and dynamic measure of individual exposure, bridging the gap left by traditional environmental monitoring. This perspective aligns with the broader goal of improving exposure assessment methodologies for more accurate and individualized health risk evaluations.

This study aimed to analyze the relationship between children’s exposure to exhaled VOCs, lung function, and residential area characteristics using advanced extraction and analytical methods for xenobiotic detection.

## 2. Materials and Methods

### 2.1. Study Design and Population

We conducted a cross-sectional study from April first, 2019, to November thirtieth, 2019, enrolling 126 asthmatic children aged 7 to 20 years from schools in Changhua County, Taiwan. Informed consent was obtained from participants and their parents. The diagnosis of asthma was determined by a physician based on symptoms, signs, pulmonary function test, and treatments by the UK National Institute of Health and Care Excellence (NICE) guideline [21]. Symptoms considered included wheezing, shortness of breath, chest tightness, and cough, particularly if these were worse at night or early morning. Pulmonary function tests, such as spirometry, were used to confirm airflow obstruction, with reversibility assessed after bronchodilator administration. Diagnostic criteria included an increase in forced expiratory volume in one second (FEV_1_) of ≥12% and ≥200 mL from baseline, consistent with asthma. Treatments prescribed, including inhaled corticosteroids or bronchodilators, further supported the diagnosis. This comprehensive approach ensures adherence to internationally recognized standards for diagnosing asthma. We collected basic information via parent-completed questionnaires, including demographics (children’s age, sex, and body mass index (BMI)), allergy history, passive smoking and insecticides exposure, and lifestyle factors. Pulmonary function was measured, and exhaled VOCs were analyzed using gas chromatography/mass spectrometry (GC-MS). The study was approved by the International Review Board of National Taiwan University Hospital (No. 20190244RIND), and written informed consent was obtained from all participants.

The subject selection process is illustrated in Supplementary Figure S1. In brief, among the 126 children residing in Changhua County, Taiwan, 5 were excluded due to unclear or unrecorded addresses, 11 had addresses that could not be validated by TGOS, and 13 lacked VOC information from GC-MS or had missing pulmonary function test data. The excluded children did not differ from the remaining 97 children included in the subsequent analysis in terms of demographics, allergy history, passive smoking and insecticide exposure, or lifestyle factors. The geographical distribution of the 97 children’s residences in Changhua County is shown in Supplementary Figure S2.

### 2.2. Pulmonary Function Test

Pulmonary function tests were conducted according to the guidelines of the American Thoracic Society and the European Respiratory Society [22]. We used the Spirolab III device (Medical International Research, Rome, Italy) for pulmonary function tests due to its portability and compliance with ATS/ERS guidelines for spirometry, making it suitable for field-based measurements. The research equipment underwent annual routine maintenance. According to the manufacturer’s user manual, the turbine sensor of the Spirolab III is designed to be calibration-free, owing to its technical features, which ensure measurement accuracy and repeatability. If significant deviations or suspected errors in measurements were observed, we arranged for repeat pulmonary function tests at the hospital that were confirmed by physicians. We obtained forced vital capacity (FVC), forced expiratory volume in one second (FEV_1_), the FEV_1_/FVC ratio, and maximum mid-expiratory flow (MMEF). A decrease in FEV_1_ and the FEV_1_/FVC ratio indicates airway obstruction, with a normal FEV_1_/FVC ratio generally being greater than 90% in children. MMEF is defined as the forced expiratory flow between 25% and 75% of the lung’s total capacity [23].

### 2.3. Collection of Breath

We designed a breath collection device equipped with an activated carbon gas filter (Spacciani Spa, Origgio, Italy) to minimize environmental VOC contamination [24,25]. The device also included a silica gel trap to reduce humidity effects and a flow restrictor (Model 7100R-R200, Hans Rudolph, Shawnee, KS, USA) to maintain a flow rate of 6 L/min. A mainstream CO_2_ monitor (EMMA Emergency Capnograph, Masimo, CA, USA) was used to monitor CO_2_ concentration, collecting alveolar air when CO_2_ levels peaked. The air was stored in FlexFoil Plus gas sampling bags (SKC Inc., Eighty Four, PA, USA), which are designed for VOC analysis and provide reliable storage for ppb-level VOCs.

### 2.4. GC-MS Analysis

One liter of breath was stored in a Bottle-Vac can (Entech Instruments Inc., Simi Valley, CA, USA) and analyzed within 48 h at the Green Energy and Environment Research Laboratory, Industrial Technology Research Institute. Analysis used an Entech 7500A auto-sampler with an Entech 7150 concentrator and an Agilent 6890 N GC/5975C MS (Agilent Technologies, Santa Clara, CA, USA). The collection of VOCs was carried out with the Solid Phase Micro Extraction (SPME) technique using a general-purpose fiber (divinylbenzene/Carboxen/PDMS). VOCs were separated with an Agilent J&W DB-1 column and analyzed by the EPA TO-15 method [26]. The EPA TO-15 method is a widely recognized analytical procedure established by the U.S. Environmental Protection Agency (EPA) for the identification and quantification of VOCs in ambient air. This method is essential in environmental monitoring, industrial hygiene, and regulatory compliance. TO-15 utilizes GC-MS, a powerful analytical technique that provides high sensitivity and specificity for VOC analysis. The method targets a wide range of VOCs, including hazardous air pollutants like benzene, toluene, and formaldehyde, with detection limits often in the parts-per-billion (ppb) range. One key advantage of TO-15 is its applicability for monitoring VOCs in various settings, such as industrial areas [27], urban environments [28], and indoor spaces, providing critical data for assessing air quality and health risks. Additionally, it plays a crucial role in identifying sources of pollution, evaluating the effectiveness of remediation efforts, and ensuring compliance with air quality standards. Calibration involved adding a standard mixture of bromochloromethane, 1,4-difluorobenzene, chlorobenzene-d5, and 1-bromo-3-fluorobenzene. Quantification was based on 63 VOC standards from the TO-15 method.

### 2.5. VOC Data Preprocessing and Validation

In quantitative analysis, raw GC-MS data were preprocessed using MSD ChemStation software (https://www.agilent.com.cn/zh-cn/support/software-informatics/massspec-workstations/gc-msd-chemstation-software/msdproductivitychemstationrev) (Agilent Technologies, Santa Clara, CA, USA). Compound identification used the National Institute of Standards and Technology (NIST) 11 database (NIST/EPA/NIH Mass Spectral Library, 2011 edition), following previously published methods [24]. Each compound was identified by matching ion chromatograms with peaks, fragments, and retention indices in the NIST11 database. Peak accuracy was verified by (1) setting the matching factor to 60% to assess fit between sample and reference spectra; (2) comparing isothermal Kovats Retention Index to identify volatile compounds, where t_n_ and t_n+1_ are the retention times of n-alkanes before and after compound X, and t_x_ is the retention time of compound X; and (3) verifying retention indices if peak comparisons showed over 75% consistency with NIST standards. Normalization is often necessary when comparing data across samples with variations in sample size, instrument conditions, or external factors influencing concentrations. Log transformation, on the other hand, is beneficial for addressing skewed distributions of VOC concentrations, as it stabilizes variance and enhances the fit of statistical models for analyses such as regression or correlation. Following the TO-15 method, VOC data normalization was performed by scaling ion abundances to *m*/*z* 95, the nominal base peak. Additionally, VOC concentrations were log-transformed to correct right-skewed distributions, as outlined in the metabolomics data-preprocessing methods described by Sun and Xia (2024) [29]. Finally, the study included VOC categories exhaled by at least 10 children, identifying 44 VOCs: 2 alcohols, 9 alkanes, 11 benzenes, 3 esters, 8 ethenes, 2 ethers, 3 freons, 4 ketones, carbon disulfide, and naphthalene. Values below the detection limit or with limits under 60 were replaced with half of the minimum dataset value [30].

### 2.6. Land Use Variables Collection

This study evaluated land use variables surrounding each child’s residential address. The residential addresses of the children were geocoded using the Taiwan Geospatial One Stop (TGOS) web resource. The land use variables included high-density and low-density residential areas, industrial areas, urban green areas, semi-natural and forested areas, and areas of all roads and major roads, with data sourced from the National Land Survey and Mapping Center. Additionally, road lengths for all roads and major roads were incorporated, with data provided by the Ministry of Transportation and Communications. These variables were processed using Quantum GIS 3.28.4 (QGIS), with different buffer sizes (i.e., circular areas around the subjects’ addresses) created at 25, 50, 100, 300, 500, and 1000 m. Distances to the nearest road (including all roads and major roads) were also calculated and included as variables to account for the influence of proximity to roads.

### 2.7. Statistical Analysis

Apart from the missing values in the VOC data, which were handled based on recommendations from previous studies [30], the questionnaire variables, land use data, and pulmonary function test results for the remaining 97 children were complete and used for subsequent analysis. Partial correlation assessed the relationships between land use variables (treated as continuous variables), VOC concentrations (continuous variables), and pulmonary function indicators (continuous variables), controlling for children’s age (continuous variable), sex, BMI (continuous variable), passive smoking exposure (category variable), and insecticide use (category variable) in the home. Two models were constructed for significant land use variables and VOCs. Model 1 evaluated the joint effects of exhaled VOCs and land use variables on pulmonary function using principal component analysis to identify components and their impact. Model 2 examined the combined effects of precursor organic gases and land use variables, using factor analysis to identify latent VOC sources and their relationships with land use variables and pulmonary function. ROC curves and AUC assessed the diagnostic ability of VOCs for pulmonary function abnormalities, with critical thresholds determined using Youden’s J statistic (sensitivity + specificity − 1) [31]. This approach was also used to identify critical land use variables (areas) affecting VOC concentrations. Data analyses were performed using SAS Version 9.4 (SAS Institute, Cary, NC, USA), and plotting was performed using R-4.3.2 software.

## 3. Results

The subject selection process is illustrated in Supplementary Figure S1. In brief, of the 126 children residing in Changhua County, Taiwan, 5 had addresses that were not clearly defined, 11 had addresses that failed for TGOS, and 13 lacked VOC information from GC-MS or had missing pulmonary function tests. Ultimately, 97 children remained for subsequent analysis. The geographical distribution of the 97 children’s residences in Changhua County is shown in Supplementary Figure S2.

The baseline characteristics and pulmonary function test results of the 97 children are summarized in Supplementary Table S1. Boys outnumbered girls, comprising 75.3% of the cohort. The average age of the children was 12.5 years. Pulmonary function test results showed that the children’s forced vital capacity (FVC) ranged from 70.0% to 124.4%, with a mean of 92.1%; forced expiratory volume in 1 s (FEV_1_) ranged from 57.7% to 127.6%, with a mean of 91.5%; FEV_1_/FVC ratio ranged from 62.3% to 97.5%, with a mean of 83.3%; and maximum mid-expiratory flow (MMEF) ranged from 30.5% to 260.7%, with a mean of 81.3%. The subjects in our study were schoolchildren who had been referred to the hospital and diagnosed with asthma based on abnormal results from school physical examinations, followed by confirmation and subsequent treatment at the hospital. By the time they were recruited for breath analysis, these children were under regular follow-up and did not necessarily exhibit active asthma symptoms or abnormal lung function. Consequently, pulmonary function test results during the recruitment period may reflect a state of controlled asthma rather than the initial diagnostic values. This explains why the reported values of FEV_1_/FVC ratio, FEV_1_, and MMEF fall within or near the normal range in the results section. The average area or length of land use variables within buffers ranging from 25 m to 1000 m around each child’s home is summarized in Supplementary Table S2. Generally, as the buffer distance increased (covering a larger area), the land use area or length also increased. The concentrations of exhaled VOCs measured by GC-MS are summarized in Table 1. The ten highest mean concentrations of exhaled VOCs for children were ethanol (54.86 ppb), isopropyl alcohol (34.61 ppb), methyl butyl ketone (21.24 ppb), 1,2-dichloroethane (4.92 ppb), 2-butanone (3.71 ppb), vinyl acetate (3.41 ppb), acetone (3.23 ppb), hexane (3.03 ppb), ethyl acetate (2.86 ppb), and dichloromethane (1.51 ppb).

**Figure 1:**
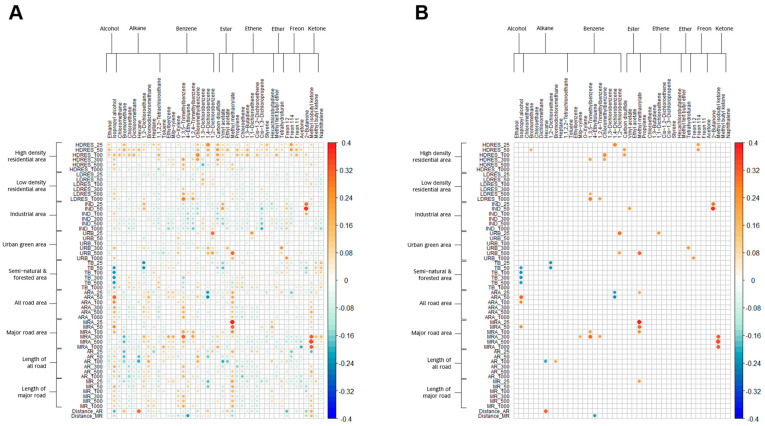
Heatmap depicting the partial correlation coefficients between land use variables and exhaled volatile organic compounds in 97 children, controlling for age, sex, body mass index, passive smoking exposure, and insecticide use in the home. (**A**) Overall correlation coefficients. (**B**) Those with statistical significant, *p* < 0.05. On the right side of the Y-axis, −0.4 to 0.4 represents the correlation coefficient. Orange-red indicates a positive correlation, while blue indicates a negative correlation. The deeper the color and the larger the circle, the stronger the correlation.

The partial correlations between land use variables and levels of exhaled VOCs are presented in Figure 1 (Figure 1A shows the overall correlation coefficients, and Figure 1B shows only those coefficients that reached statistical significance). It can be seen that high-density residential areas near the home are generally positively correlated with VOC concentrations, with significant correlations observed for bromomethane, 1,3,5-trimethylbenzene, chloromethylbenzene, 1,4-dichlorobenzene, carbon disulfide, and freon 114. The correlations between low-density residential areas near the home and VOCs were weaker, with significant positive correlations observed only for 1,3,5-trimethylbenzene and 1,2,4-trimethylbenzene at buffer distances greater than 500 m. Industrial areas near the home showed mostly negative correlations with VOCs (blue), though none reached statistical significance, except for vinyl acetate and 2-butanone, which showed significant positive correlations within 50 m. Urban green areas near the home had weak correlations with most VOCs, but significant positive correlations were observed for 1,2-dichlorobenzene, methyl methacrylate, 1,1-dichloroethene, tetrahydrofuran, and freon 12. Semi-natural and forest areas near the home showed significant negative correlations with isopropyl alcohol and 1,2-dichloroethane. All road areas within 300 m of the home showed significant positive correlations with isopropyl alcohol and methyl methacrylate, but significant negative correlations with 1,4-dichlorobenzene. Major road areas near the home showed significant positive correlations with isopropyl alcohol, m/p-xylene, 1,3,5-trimethylbenzene, 1,2,4-trimethylbenzene, methyl methacrylate, and methyl isobutyl ketone. The correlation with methyl methacrylate weakened as the buffer distance increased from 25 m to 100 m; the correlations with 1,3,5-trimethylbenzene and methyl isobutyl ketone strengthened as the buffer area covering the major road increased. The length of all roads or major roads near the home was significantly positively correlated with only a few VOC concentrations. The proximity to the nearest all road or major road showed similar patterns.

Supplementary Figure S3 presents detailed heatmaps showing the correlation coefficients between land use variables, exhaled VOCs, and pulmonary function test indicators. Most land use variables had weak negative correlations with pulmonary function, while urban green and semi-natural areas had weak positive correlations. High-density residential areas were significantly negatively correlated with FVC, and industrial areas were positively correlated with MMEF. Semi-natural and forest areas had significant positive correlations with FVC and FEV_1_. Most VOCs had negative correlations with FVC and FEV_1_, but positive correlations with FEV_1_/FVC and MMEF. Significant correlations with pulmonary function tests were observed for 1,3-dichlorobenzene and tetrahydrofuran.

This study further modeled and described the complex relationships between land use variables, VOC levels, and pulmonary function. Model 1 constructed the correlations between the principal components of land use variables and VOC levels with pulmonary function (Supplementary Figure S4). A total of 15 principal components with eigenvalues greater than 1 were selected, collectively explaining 78.5% of the variance in VOC levels (Supplementary Figure S5). The variable loadings of each principal component are summarized in Supplementary Table S3, and the correlations between the 15 principal components and pulmonary function are presented in Supplementary Figure S6. Only the second principal component showed a significant negative correlation with FEV_1_/FVC. The major road area within 25 to 300 m of the home had the primary positive loading for land use variables in the second principal component (coefficients ranging from 0.200 to 0.241), while VOCs such as methyl methacrylate (coefficient: 0.119), 1,3,5-trimethylbenzene (coefficient: 0.090), 1,2,4-trimethylbenzene (coefficient: 0.087), and m/p-xylene (coefficient: 0.076) had the main positive loadings (Supplementary Table S3).

We also attempted another model to describe the relationships between land use variables, VOC levels, and pulmonary function (Supplementary Figure S7): the relationships between VOCs, land use variables, and pulmonary function were derived from the precursor mixtures of VOCs. Under this model, VOCs were identified using five factors (precursor mixtures) (Table 2). The loadings of the 10 principal components identified from the five factors and land use variables are summarized in Supplementary Table S4. The correlations between the 10 principal components and pulmonary function (Supplementary Figure S8) show a significant negative correlation between the second principal component and the FEV_1_/FVC. The major road area within 25 to 300 m of the home had the primary positive loading for land use variables in the second principal component (coefficients ranging from 0.232 to 0.293). Additionally, Factor 1 also had a primary positive loading in the second principal component, while Factor 2 had a primary negative loading (Supplementary Table S4). Among the VOCs with loadings in the same direction as the factors, m/p-xylene (Factor 1’s coefficient, 0.543; Factor 2’s coefficient, −0.391), 1,3,5-trimethylbenzene (Factor 1’s coefficient, 0.657; Factor 2’s coefficient, −0.538), and 1,2,4-trimethylbenzene (Factor 1’s coefficient, 0.637; Factor 2’s coefficient, −0.579) were identified as the major contributors (Table 2).

This study also evaluated the diagnostic performance of m/p-xylene, 1,3,5-trimethylbenzene, and 1,2,4-trimethylbenzene individually in predicting abnormal FEV_1_/FVC (<median (83.7%) or <90%). The ROC curves are presented in Supplementary Figure S9, and the corresponding model coefficients are shown in Supplementary Table S5. The AUC for predicting FEV_1_/FVC < median (83.7%) using each of the three VOCs individually ranged from 0.55 to 0.57, which was not statistically significant. Similarly, the AUCs for predicting FEV_1_/FVC < 90% ranged from 0.54 to 0.59, also not statistically significant. However, when all three VOCs were used together to predict FEV_1_/FVC < median, the AUC was 0.65 (95% Wald CI: 0.54 to 0.75). The combined VOCs showed better predictive performance for FEV_1_/FVC < 90%, with an AUC of 0.66 (95% Wald CI: 0.53 to 0.79) (Supplementary Figure S10 and Supplementary Table S6).

**Figure 2:**
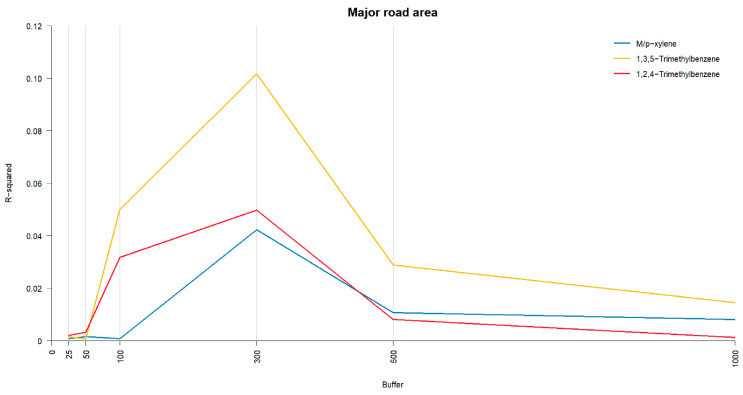
The R-squared between M/p-xylene, 1,3,5-trimethylbenzene, 1,2,4-trimethylbenzene, and major road area.

The explanatory power of m/p-xylene, 1,3,5-trimethylbenzene, and 1,2,4-trimethylbenzene was found to be highest within a 300 m buffer of major road areas (Figure 2). Under the abnormal FEV_1_/FVC% criteria (<median (83.7%) or <90%), we were able to delineate the high-risk levels of VOCs (according to predicted probability for abnormal FEV_1_/FVC% criteria) and further identify the potential threshold areas for major roads within a 300 m buffer (Table 3). Specifically, for residences within 300 m of a major road area exceeding 14,170.52 square meters, the probability of FEV_1_/FVC falling below the median (83.7%) increased significantly. Additionally, for residences within 300 m of a major road area exceeding 275.8 square meters, the probability of FEV_1_/FVC falling below 90% was significantly higher.

## 4. Discussion

In this study, we used appropriate extraction and analytical methods for separation and determination of mixtures of xenobiotics, including collecting alveolar air samples, solid phase microextraction of xenobiotics, and standardized methods to process the metabolomics data. Our study was then able to analyze the relationship between children’s exposure to exhaled VOCs and lung function and children’s residential areas (land use). To our knowledge, no previous research has simultaneously investigated the interplay between these two factors. This study, conducted in Changhua County, Taiwan, involving 97 asthmatic children, found that exhaled 1,3,5-trimethylbenzene, 1,2,4-trimethylbenzene, and m/p-xylene were associated with the area of major roads within a 300 m buffer of their residences and a decreased FEV_1_/FVC ratio. Specifically, when the area of major roads exceeded 275.8 square meters, there was an increased risk of elevated levels of these three VOCs and a decline in the FEV_1_/FVC ratio below 90%. As anthropogenic aromatic hydrocarbons, 1,3,5-trimethylbenzene, 1,2,4-trimethylbenzene, and m/p-xylene can originate from both indoor sources [32,33] and outdoor sources (e.g., road, vehicle exhaust, gas stations, and industrial emissions) [34,35,36,37]. These support our findings.

Additionally, some studies suggest that proximity to major roads within 50 or 100 m is more strongly associated with respiratory diseases than the 300 m distance observed in our study [38,39,40,41]. In fact, our results highlight the importance of major road area exceeding a certain threshold (e.g., over 275.8 square meters). The 300 m distance likely reflects that most children in this study lived farther from major roads, until the area within this range was sufficient to establish a significant relationship with VOCs. Principal component analysis also shows a strong loading effect of major road area from 25 to 300 m on the FEV_1_/FVC ratio. Therefore, we support the importance of the major road area within 300 m, provided it meets the threshold. Previous meta-analyses also indicate that areas within 300 m of major roads are high-risk zones for air pollutants, with risk levels decreasing to background levels beyond 300 to 500 m [42]. However, further study is needed to validate the thresholds for major road distance and area associated with increased VOC exposure levels and reduced pulmonary function.

The health effects of trimethylbenzenes and xylenes are often studied in the context of exposure to mixtures of benzene isomers and other VOCs, making it difficult to determine the specific effects of 1,3,5-trimethylbenzene, 1,2,4-trimethylbenzene, and m/p-xylene. However, high concentrations of trimethylbenzene or xylene, when inhaled or contacted occupationally as organic solvents, are generally recognized to cause skin or eye irritation, neurological effects, respiratory issues, and both cancerous and non-cancerous diseases [43,44,45,46,47,48]. In children, some studies have reported associations between 1,2,4-trimethylbenzene and m/p-xylene and conditions such as asthma, rhinitis, and other allergic effects; however, these studies mainly focus on indoor exposures [49,50,51]. However, there is still limited research on how these compounds affect children’s pulmonary function, even with indoor exposure [17]. The effects of low-level inhalation of VOCs from outdoor sources on health effects or pulmonary function also remain unclear. It is known that in outdoor air, 1,3,5-trimethylbenzene, 1,2,4-trimethylbenzene, and m/p-xylene can react with hydroxyl radicals and ozone in the presence of NO_x_, forming surface ozone and secondary organic aerosols, which contribute to air pollution and pose health risks [52,53]. Whether children inhaling surface ozone experience oxidative stress in respiratory cells and tissues [54], leading to the endogenous production and release of trimethylbenzenes or xylenes [55] and ultimately affecting lung function [56], is necessary to further investigate. However, Model 2 in this study hypothesizes that the combined effect of major roads and exhaled VOC precursors (e.g., surface ozone) on children’s pulmonary function identifies three key VOCs—1,3,5-trimethylbenzene, 1,2,4-trimethylbenzene, and m/p-xylene—associated with a decrease in FEV_1_/FVC ratio.

According to a recent report by Moura et al. on exhaled VOCs as biomarkers for diagnosing diseases [57], 1,2,4-trimethylbenzene may serve as a biomarker for lung cancer [58], and 1,3,5-trimethylbenzene has been associated with sleep apnea [59] and active pulmonary tuberculosis [60,61]. However, no evidence supports the use of these two trimethylbenzenes as biomarkers for asthma or COPD. On the other hand, m-xylene and p-xylene have been linked to lung cancer [62], with p-xylene potentially indicating asthma in children [63]. Regarding the pulmonary function, a study has shown significant negative correlations between exhaled acetone, tetradecane, and pentadecane and pulmonary function parameters in infants [64]. However, acetone in our study showed no significant association with pulmonary function in children. Nevertheless, this study is the first to mention the association between exhaled 1,3,5-trimethylbenzene, 1,2,4-trimethylbenzene, and m/p-xylene in children and a decreased FEV_1_/FVC ratio, suggesting that the simultaneous detection of these three VOCs could serve as a potential biomarker for pulmonary function. Additionally, the combination of these VOCs demonstrated significant diagnostic ability in detecting an FEV_1_/FVC ratio below 90%, with an AUC of 0.66 (*p* < 0.05). It should be noted that in diagnosing asthma, an FEV_1_/FVC ratio below 90% must be accompanied by an FEV_1_ below 80% and an increase in FEV_1_ of more than 12% after bronchodilation. We acknowledge that these three compounds still have a long way to go as diagnostic markers for asthma, particularly given the low-level VOC exposure in this study. Nevertheless, this study primarily highlights the correlation between exhaled 1,3,5-trimethylbenzene, 1,2,4-trimethylbenzene, and m/p-xylene in children and the decline in pulmonary function.

In both models proposed in this study, high-density residential areas within a 25 to 100 m range showed a positive loading effect on the FEV_1_/FVC ratio, which also influenced the positive relationship between bromomethane, freon 114, and the FEV_1_/FVC ratio. However, this should not be interpreted as indicating that high-density residential areas improve pulmonary function. First, we observed that high-density residential areas within a 25 to 50 m buffer significantly reduced FVC, while the impact on FEV_1_ was relatively minor, resulting in an apparent increase in the FEV_1_/FVC ratio. Second, we observed a negative correlation between high-density residential areas and major road areas in the same buffer zones (i.e., more high-density buildings were associated with fewer major roads). This again implies that the presence of major roads plays a significant role in the decline of pulmonary function. In fact, high-density residential areas may be associated with increased levels of O_3_ or PM_2.5_ [65,66].

This study has limitations. First, as a cross-sectional study, it is hard to establish causality. However, we reviewed that the characteristics of the areas around children’s residences in Changhua County, Taiwan, from 2013 to 2019 were relatively stable. This stability allows us to consider outdoor pollutant exposure as a consistent, long-term source, maintaining a temporal relationship with VOC measurements and pulmonary function tests. Second, a limited sample size may reduce the statistical power of our findings. In spite of this, we believe our results are generalizable and reflect a reproducible association. Finally, the potential health effects of low-level VOC exposure remain an important area of investigation. While our current study focused on the analysis of VOC biomarkers in exhaled breath, we acknowledge that environmental factors such as air fresheners, insect sprays, and household materials containing solvents could contribute to random fluctuations in ambient VOC levels within residential premises [67]. Nonetheless, formaldehyde, a significant indoor volatile organic compound, was not detected in the exhaled breath of children in this study. Additionally, we controlled for main indoor VOC sources (such as passive smoking exposure and insecticide use), as well as children’s personal characteristics (such as age, sex, and BMI), which have been reported as risk factors for 1,3,5-trimethylbenzene, 1,2,4-trimethylbenzene, and m/p-xylene [68]. To strengthen the conclusions of this study, future research could incorporate measurements of initial ambient VOC levels in residential environments. This would allow us to better assess the influence of these random fluctuations and contextualize the exhaled breath VOC findings. Such an approach could also help identify potential confounding factors and enhance our understanding of the relationship between environmental VOC exposure and its biological effects.

This study emphasizes the need to not only consider VOCs as biomarkers for pulmonary function abnormalities but also to identify harmful environmental factors associated with these VOCs and offer potential risk mitigation strategies. Therefore, this study aims to identify key VOCs in children that are associated with the characteristics of their residential areas and linked to pulmonary function abnormalities. The next step is to explore the thresholds of area characteristics and VOC levels that increase the risk of pulmonary function abnormalities. These findings are expected to provide a basis for developing environmental policies and public health strategies aimed at reducing children’s exposure to VOCs and promoting respiratory health in this vulnerable population. We suggest future research to explore the feasibility of integrating immunological tests, such as measuring specific antibodies or other biomarkers related to VOC exposure. These tests could help identify individual susceptibility and provide a more comprehensive understanding of the relationship between VOC exposure and health outcomes. Such an extension would complement the geographic and environmental data already analyzed in this study and further contribute to the field of exposure science.

## 5. Conclusions

This study identified exhaled 1,3,5-trimethylbenzene, 1,2,4-trimethylbenzene, and m/p-xylene and explored their association with a reduced FEV_1_/FVC ratio in children and the major road areas near to children’s residences. This study investigated the potential of these VOCs as biomarkers for impaired pulmonary function in children, addressing a gap in research on low-level outdoor VOCs inhaled exposure and pediatric respiratory health. Future research should continue to explore and validate environmental thresholds and the potential of specific VOCs as biomarkers. This study hopes to provide a basis for addressing public health issues related to reducing children’s exposure to harmful VOCs and promoting respiratory health.

## Figures and Tables

**Table 1:** Summary statistics of exhaled volatile organic compound concentration for 97 children.

Categories of VOCs	VOCs	Number and Percentage of Children with Response		VOC Concentration (ppb) (N = 97)				
		N (% of Total 97 Children)		Mean ± SD	Median	Minimum	Maximum	IQR
Alcohol	Ethanol	81	(83.5)	54.86 ± 196.69	14.74	0.00	1741.48	3.89–38.09
	Isopropyl alcohol	97	(100.0)	34.61 ± 75.02	4.75	0.28	388.16	1.39–23.60
Alkane	Chloromethane	65	(67.0)	1.21 ± 5.64	0.16	0.00	54.62	0.00–0.71
	Bromomethane	44	(45.4)	0.17 ± 0.43	0.00	0.00	3.54	0.00–0.13
	Chloroethane	48	(49.5)	0.80 ± 3.44	0.00	0.00	33.29	0.00–0.61
	Dichloromethane	56	(57.7)	1.51 ± 10.17	0.12	0.00	99.66	0.00–0.37
	Hexane	74	(76.3)	3.03 ± 19.74	0.21	0.00	177.59	0.05–0.53
	1,2-Dichloroethane	80	(82.5)	4.92 ± 5.85	2.43	0.00	32.15	0.21–9.17
	Bromodichloromethane	10	(10.3)	0.01 ± 0.02	0.00	0.00	0.15	0.00–0.00
	Heptane	42	(43.3)	0.11 ± 0.35	0.00	0.00	2.91	0.00–0.07
	1,1,2,2-Tetrachloroethane	55	(56.7)	0.13 ± 0.46	0.03	0.00	4.19	0.00–0.10
Benzene	Toluene	17	(17.5)	0.01 ± 0.03	0.00	0.00	0.17	0.00–0.00
	Ethylbenzene	92	(94.8)	0.39 ± 1.91	0.04	0.00	16.21	0.02–0.11
	m/p-Xylene	83	(85.6)	0.40 ± 2.11	0.05	0.00	18.11	0.02–0.13
	o-Xylene	75	(77.3)	0.13 ± 0.21	0.07	0.00	1.14	0.00–0.16
	1,3,5-Trimethylbenzene	60	(61.9)	0.04 ± 0.11	0.02	0.00	0.79	0.00–0.04
	4-Ethyltoluene	73	(75.3)	0.04 ± 0.06	0.02	0.00	0.39	0.00–0.04
	1,2,4-Trimethylbenzene	69	(71.1)	0.08 ± 0.22	0.02	0.00	1.77	0.00–0.06
	Chloromethylbenzene	62	(63.9)	0.15 ± 0.39	0.04	0.00	2.61	0.00–0.12
	1,3-Dichlorobenzene	11	(11.3)	0.00 ± 0.00	0.00	0.00	0.02	0.00–0.00
	1,4-Dichlorobenzene	14	(14.4)	0.00 ± 0.01	0.00	0.00	0.03	0.00–0.00
	1,2-Dichlorobenzene	24	(24.7)	0.01 ± 0.02	0.00	0.00	0.17	0.00–0.00
Carbon disulfide	Carbon disulfide	62	(63.9)	1.07 ± 3.94	0.05	0.00	31.32	0.00–0.32
Ester	Vinyl acetate	92	(94.8)	3.41 ± 7.33	0.42	0.00	44.57	0.20–2.42
	Ethyl acetate	52	(53.6)	2.86 ± 16.41	0.09	0.00	145.12	0.00–0.49
	Methyl methacrylate	19	(19.6)	0.11 ± 0.37	0.00	0.00	2.58	0.00–0.00
Ethene	Propylene	95	(97.9)	1.37 ± 5.17	0.35	0.00	48.32	0.15–0.69
	Chloroethene	48	(49.5)	0.43 ± 2.30	0.00	0.00	22.37	0.00–0.18
	1,3-Butadiene	56	(57.7)	0.75 ± 2.96	0.11	0.00	25.03	0.00–0.54
	1,1-Dichloroethene	50	(51.5)	0.37 ± 1.49	0.04	0.00	11.38	0.00–0.14
	Cis-1,2-Dichloroethene	47	(48.5)	0.98 ± 6.92	0.00	0.00	67.90	0.00–0.21
	Cis-1,3-Dichloropropene	22	(22.7)	0.09 ± 0.28	0.00	0.00	2.12	0.00–0.00
	Styrene	72	(74.2)	0.20 ± 0.53	0.07	0.00	4.95	0.00–0.24
	Hexachlorobutadiene	43	(44.3)	0.01 ± 0.02	0.00	0.00	0.07	0.00–0.02
Ether	Methyl tert-butyl ether	86	(88.7)	0.58 ± 1.46	0.25	0.00	13.72	0.08–0.56
	Tetrahydrofuran	20	(20.6)	0.84 ± 7.39	0.00	0.00	72.87	0.00–0.00
Freon	Freon 12	23	(23.7)	0.01 ± 0.04	0.00	0.00	0.21	0.00–0.00
	Freon 114	30	(30.9)	0.04 ± 0.18	0.00	0.00	1.64	0.00–0.02
	Freon 11	51	(52.6)	0.19 ± 0.76	0.02	0.00	6.98	0.00–0.09
Ketone	Acetone	90	(92.8)	3.23 ± 6.64	0.88	0.00	42.05	0.28–2.35
	2-Butanone	97	(100.0)	3.71 ± 11.48	0.65	0.07	100.58	0.23–2.40
	Methyl isobutyl ketone	60	(61.9)	0.47 ± 0.99	0.07	0.00	4.92	0.00–0.27
	Methyl butyl ketone	85	(87.6)	21.24 ± 121.73	1.01	0.00	862.65	0.38–3.70
Naphthalene	Naphthalene	34	(35.1)	0.04 ± 0.15	0.00	0.00	1.27	0.00–0.03

Abbreviation: VOCs, volatile organic compounds; SD, standard deviation; IQR, interquartile range.

**Table 2:** The loading of factors among 21 exhaled volatile organic compounds.

VOCs	Factor1	Factor2	Factor3	Factor4	Factor5
Isopropyl alcohol	0.219				
Bromomethane		0.721		−0.235	
Hexane				−0.249	0.616
1,2-Dichloroethane		−0.294	0.630		
Bromodichloromethane	−0.131				
M/p-xylene	0.543	−0.391			
1,3,5-Trimethylbenzene	0.657	−0.538			
4-Ethyltoluene	0.346	−0.296			
1,2,4-Trimethylbenzene	0.637	−0.579			
Chloromethylbenzene	0.127				−0.027
1,4-Dichlorobenzene		0.101	−0.260		
1,2-Dichlorobenzene			−0.367	0.392	
Carbon disulfide	0.701			−0.219	
Vinyl acetate	0.608				−0.088
Methyl methacrylate		−0.115	0.123		
1,1-Dichloroethene			−0.119	0.514	
Tetrahydrofuran				−0.251	0.562
Freon 12		0.058	−0.267		
Freon 114		0.709		−0.202	
2-Butanone				0.539	−0.065
Methyl isobutyl ketone	0.373			−0.371	

Abbreviation: VOCs, volatile organic compounds.

**Table 3:** Under the abnormal FEV_1_/FVC% criteria associated with the risk level of exhaled volatile organic compounds, the threshold for major road areas within a 300-m buffer.

	VOCs			Major Road Area with Buffer 300 m (MRA_300)			
Abnormal FEV_1_/FVC% Criteria	Model	AUC	Predicted Probability (Maximum of Youden Index = se + sp − 1)	Model	AUC	Predicted Probability (Maximum of Youden Index)	Threshold (Area, m^2^)
FEV_1_/FVC% < median	Logit(P(FEV_1_/FVC% < Median)) = 3 VOCs (categories)	0.65	0.54 (0.43 + 0.77 − 1 = 0.20)	Logit(P(3 VOCs ≥ 0.54)) = MRA_300 (continuous)	0.51	0.35 (0.05)	14,170.5
FEV_1_/FVC% < 90%	Logit(P(FEV_1_/FVC% < 90%)) = 3 VOCs (categories)	0.66	0.80 (0.71 + 0.55 − 1 = 0.26)	Logit(P(3 VOCs ≥ 0.80)) = MRA_300 (continuous)	0.58	0.53 (0.21)	275.8

Abbreviation: VOCs, volatile organic compounds; FEV_1_, forced expiratory volume in one second; FVC, forced vital capacity; AUC, area under the receiver operating characteristic curves; MRA_300, major road area with buffer 300 m; se, sensitivity; sp, specificity.

## Data Availability

The original contributions presented in this study are included in the article/Supplementary Materials. Further inquiries can be directed to the corresponding author.

## Supplementary Materials

The following supporting information can be downloaded at https://www.mdpi.com/article/10.3390/jox15010027/s1, Supplementary Table S1: Characteristics of 97 children and their pulmonary function test results; Supplementary Table S2: The mean and standard deviation of the area of land use variables within a buffer size of 25 to 1000 m from the residences of the children; Supplementary Table S3: The loading table of 15 principal components from Model 1; Supplementary Table S4: The loading table of 10 principal components, including 5 factors, from Model 2; Supplementary Table S5: Predicted model for abnormal FEV_1_/FVC% (<median or <90%) based on m/p-xylene, 1,3,5-trimethylbenzene, and 1,2,4-trimethylbenzene, respectively; Supplementary Table S6: Predicted model for abnormal FEV_1_/FVC% (<median or <90%) considering simultaneously m/p-xylene, 1,3,5-trimethylbenzene, and 1,2,4-trimethylbenzene; Supplementary Figure S1: Flowchart of study subjects; Supplementary Figure S2: The geographical location of the 97 children’s residences in Changhua County, Taiwan; Supplementary Figure S3: Heatmap depicting the correlation coefficients between pulmonary function test and (A) land use variables and (B) exhaled volatile organic compounds of 97 children. (The heatmap on the left of (A) and (B) presents overall correlation coefficients; the heatmap on the right is presented those with statistical significance, *p* < 0.05); Supplementary Figure S4: Model 1 assuming the relationship between the land use (LU) variables, the exhaled volatile organic compounds (VOCs), and the pulmonary function test. (PC, principal components); Supplementary Figure S5: The scree plot and the variance explained plot from the result of principal components analysis from Model 1; Supplementary Figure S6: Heatmap depicting the correlation coefficients between 15 principal components and the pulmonary function test: (A) overall correlation coefficients; (B) those with statistical significant, *p* < 0.05; Supplementary Figure S7: Model 2 assuming the relationship between the land use (LU) variables, the precursors (factors) of the exhaled volatile organic compounds (VOCs), and the pulmonary function test. (FA, factor; PC, principal component; e, measurement errors.); Supplementary Figure S8: Heatmap depicting the correlation coefficients between the 10 principal components and the pulmonary function test: (A) overall correlation coefficients; (B) those with statistical significant, *p* < 0.05; Supplementary Figure S9: Receiver operating characteristic (ROC) curves for the abnormal FEV_1_/FVC% criteria (<median (83.7%) or <90%) based on m/p-xylene (categories: ≤0.01, 0.02–0.03, 0.04–0.07, 0.08–0.16, and 0.17+ ppb), 1,3,5-trimethylbenzene (categories: ≤0.01, 0.02–0.03, 0.04–0.05, and 0.06+ ppb), and 1,2,4-trimethylbenzene (categories: ≤0.01, 0.02–0.03, 0.04–0.08, and 0.09+ ppb), respectively; Supplementary Figure S10: Receiver operating characteristic (ROC) curves for the abnormal FEV_1_/FVC% criteria (<median (83.7%) or <90%) considering simultaneously m/p-xylene (categories: ≤0.01, 0.02–0.03, 0.04–0.07, 0.08–0.16, and 0.17+ ppb), 1,3,5-trimethylbenzene (categories: ≤0.01, 0.02–0.03, 0.04–0.05, and 0.06+ ppb), and 1,2,4-trimethylbenzene (categories: ≤0.01, 0.02–0.03, 0.04–0.08, and 0.09+ ppb).
