# Supplementary material for: Residential Proximity Land Use Characteristics and Exhaled Volatile Organic Compounds’ Impact on Pulmonary Function in Asthmatic Children
Source: J Xenobiot. 2025 Feb 5;15(1):27. doi: 10.3390/jox15010027 (PMC11856375; doi:10.3390/jox15010027)
Supplement: Supplementary file 1 [file jox-15-00027-s001.zip › jox-3358494-supplementary.pdf]

Supplementary Materials

Supplementary Table:

Supplementary Table S1. Characteristics of 97 Children and Their Pulmonary Function Test Results.

Supplementary Table S2. The Mean and Standard Deviation of the Area of Land Use Variables within a Buffer Size of 25 to 1000 Meters from the Residences of the Children.

Supplementary Table S3. The Loading Table of 15 Principal Components from Model 1.

Supplementary Table S4. The Loading Table of 10 Principal Components Including 5 Factors from Model 2.

Supplementary Table S5. Predicted Model for Abnormal $FEV_1/FVC\%$ (<Median or <90%) Based on M/p−Xylene, 1,3,5−Trimethylbenzene, and 1,2,4−Trimethylbenzene, respectively.

Supplementary Table S6. Predicted Model for Abnormal $FEV_1/FVC\%$ (<Median or <90%) Considering Simultaneously M/p−Xylene, 1,3,5−Trimethylbenzene, and 1,2,4−Trimethylbenzene.

Supplementary Figure:

Supplementary Figure S1. Flowchart of Study Subjects.

Supplementary Figure S2. The Geographical Location of the 97 Children's Residences in Changhua County, Taiwan.

Supplementary Figure S3. Heatmap Depicting the Correlation Coefficients between Pulmonary Function Test and A) Land Use Variables, and B) Exhaled Volatile Organic Compounds of 97 Children. (The Heatmap on the Left of A and B is Presented Overall Correlation Coefficients; the Heatmap on the Right is Presented Those with Statistical Significant, $p<0.05$)

Supplementary Figure S4. Model 1 Assuming the Relationship between the Land Use Variables (LU), the Exhaled Volatile Organic Compounds (VOCs), and the Pulmonary Function Test. (PC, principal components)

Supplementary Figure S5. The Scree Plot and the Variance Explained Plot from the Result of Principal Components Analysis from Model 1.

Supplementary Figure S6. Heatmap Depicting the Correlation Coefficients between 15 Principal Components and the Pulmonary Function Test. (A, Overall Correlation Coefficients; B, Those with Statistical Significant, $p<0.05$)

Supplementary Figure S7. Model 2 Assuming the Relationship between the Land Use Variables (LU), the Precursors (Factors) of the

Exhaled Volatile Organic Compounds (VOCs), and the Pulmonary Function Test. (FA, Factor; PC, principal component; e, measurement errors)

Supplementary Figure S8. Heatmap Depicting the Correlation Coefficients between the 10 Principal Components and the Pulmonary Function Test. (A, Overall Correlation Coefficients; B, Those with Statistical Significant, $p<0.05$)

Supplementary Figure S9. Receiver Operating Characteristic Curves (ROC) for the Abnormal FEV_1/FVC% Criteria (<Median (83.7%) or <90%) Based on M/p−Xylene (Categories: ≤ 0.01, 0.02−0.03, 0.04−0.07, 0.08−0.16, 0.17+ ppb), 1,3,5−Trimethylbenzene (Categories: ≤ 0.01, 0.02−0.03, 0.04−0.05, 0.06+ ppb), and 1,2,4−Trimethylbenzene (Categories: ≤0.01, 0.02−0.03, 0.04−0.08, 0.09+ ppb), respectively.

Supplementary Figure S10. Receiver Operating Characteristic (ROC) Curves for the Abnormal FEV_1/FVC% Criteria (<Median (83.7%) or <90%) Considering Simultaneously M/p−Xylene (Categories: ≤ 0.01, 0.02−0.03, 0.04−0.07, 0.08−0.16, 0.17+ ppb), 1,3,5−Trimethylbenzene (Categories: ≤ 0.01, 0.02−0.03, 0.04−0.05, 0.06+ ppb), and 1,2,4−Trimethylbenzene (Categories: ≤0.01, 0.02−0.03, 0.04−0.08, 0.09+ ppb).

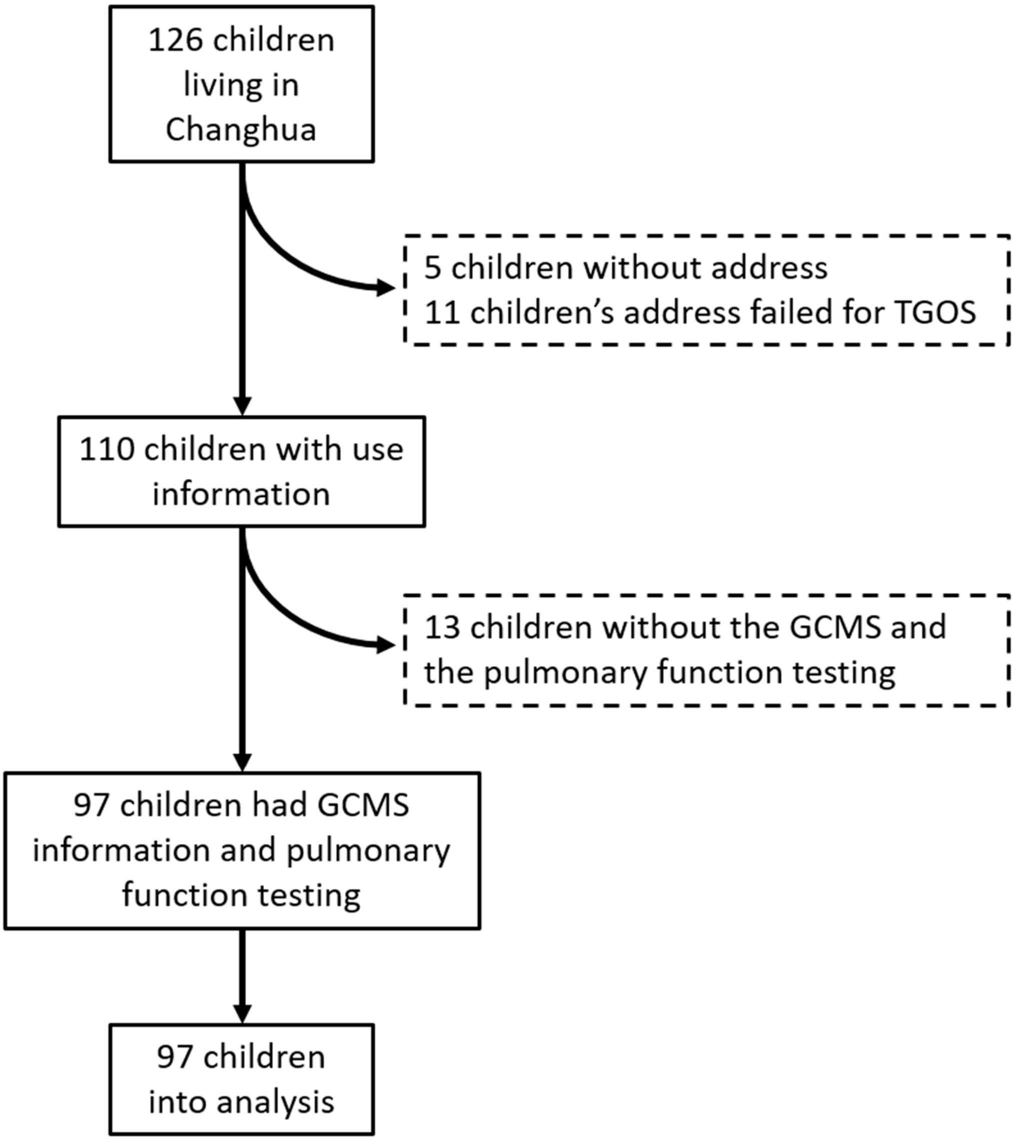

Supplementary Figure S1. Flowchart of Study Subjects.

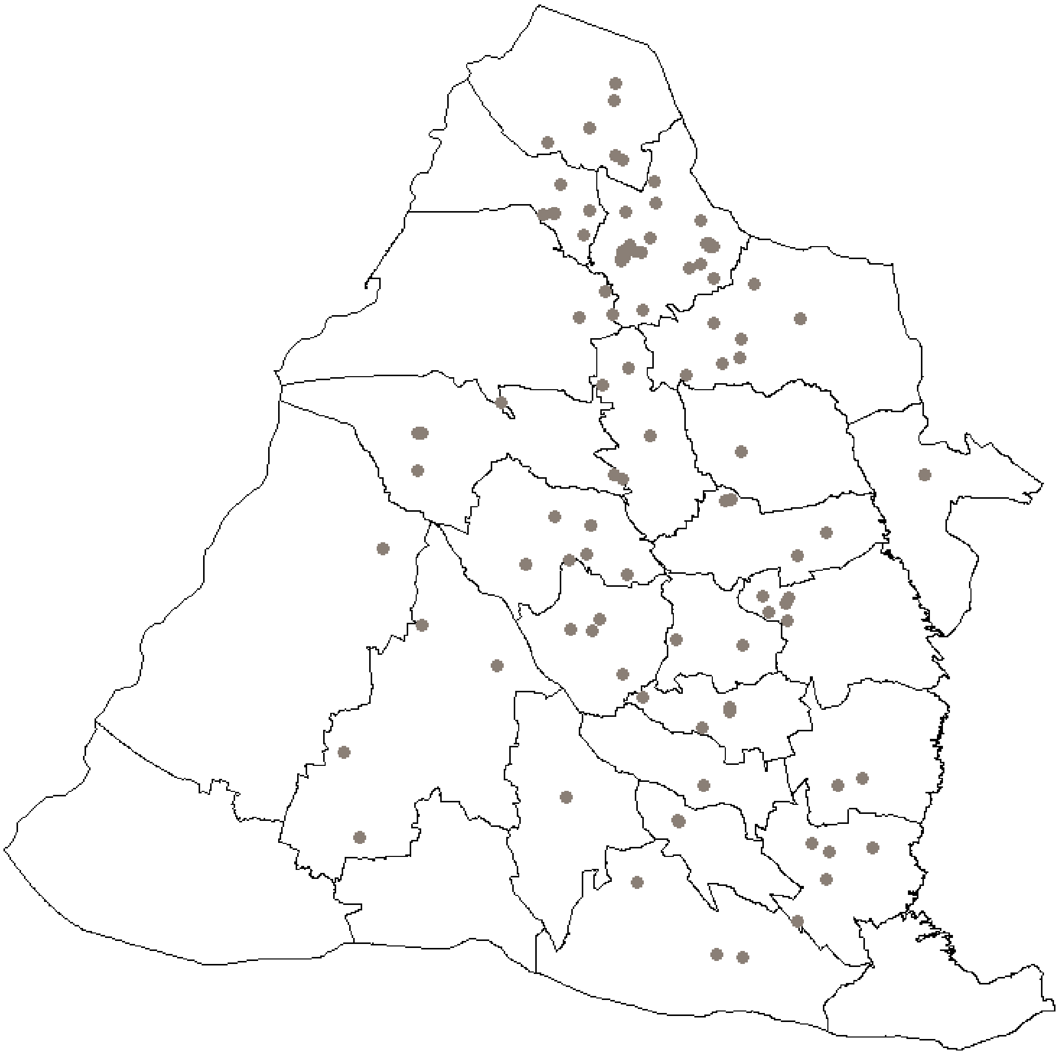

Supplementary Figure S2. The Geographical Location of the 97 Children's Residences in Changhua County, Taiwan.

Supplementary Table S1. Characteristics of 97 Children and Their Pulmonary Function Test Results.

**Characteristics/ Pulmonary Function Test**	**Total Children (N=97)**	**%**
Sex		
Girls	24	24.7
Boys	73	75.3
Age, Mean ± SD, year	12.5 ± 1.9	
7-10	12	12.6
11-15	81	85.3
16-20	2	2.1
Body mass index (BMI), Mean ± SD, kg/m^2	22.1 ± 5.1	
<15.4	8	8.3
15.4-21.4	33	34.0
21.5-23.9	20	20.6
≥ 24.0	31	32.0
Missing	5	5.1
Passive smoking exposure		
No	42	43.3
Yes	55	56.7
Use of insecticides		
No	82	84.5
Yes	15	15.5
Pulmonary function test		
Forced vital capacity (FVC), %		
Mean ± SD	92.1 ± 11.8	
Minimum	70.0	
Maximum	124.4	
Forced expiratory volume in the 1 second (FEV_1), %		
Mean ± SD	91.5 ± 11.9	
Minimum	57.7	
Maximum	127.6	
FEV_1/FVC, %		
Mean ± SD	83.3 ± 7.7	
Minimum	62.3	
Maximum	97.5	
Maximum mid-expiratory flow (MMEF), %		
Mean ± SD	81.3 ± 29.6	
Minimum	30.5	
Maximum	260.7	

Abbreviation: SD, standard deviation

Supplementary Table S2. The Mean and Standard Deviation of the Area of Land Use Variables within a Buffer Size of 25 to 1000 Meters from the Residences of the Children.

**Land use variables (buffer, meters)**	**Area (m^2) / Length (m) (N=97)**			
	**Mean ± SD**	**Minimum**	**Median**	**Maximum**
High density residential area (HDRES)				
25	1024.6 ± 439.7	128.3	1034.4	1981.5
50	3294.3 ± 1529.6	330.7	3314.1	7482.4
100	10457.3 ± 5158.3	918.9	10203.5	21990.8
300	56102.1 ± 22579.0	4666.0	55577.0	108273.1
500	123477.9 ± 53226.0	8566.7	110905.2	278384.2
1000	392475.7 ± 178216.7	22749.0	364103.8	816613.0
Low density residential area (LDRES)				
25	99.2 ± 218.6	0.0	0.0	1093.6
50	327.5 ± 607.3	0.0	75.8	3666.5
100	986.4 ± 1623.0	0.0	258.1	8115.3
300	6089.5 ± 8482.8	0.0	2860.1	39832.1
500	15042.1 ± 20396.5	0.0	6225.3	90027.1
1000	50723.6 ± 66744.1	0.0	20996.4	320708.5
Industrial area (IND)				
25	39.4 ± 102.8	0.0	0.0	565.1
50	282.2 ± 514.7	0.0	0.0	2654.3
100	1592.8 ± 2340.1	0.0	605.9	11108.2
300	15457.4 ± 18384.6	0.0	8778.1	91556.6
500	45949.2 ± 47913.0	0.0	29983.5	251825.3
1000	183720.9 ± 132151.1	2267.8	161007.6	555361.6
Urban green area (URB)				
25	11.5 ± 57.0	0.0	0.0	371.7
50	39.3 ± 235.2	0.0	0.0	2219.3
100	165.4 ± 638.8	0.0	0.0	5497.9
300	1858.1 ± 3784.9	0.0	550.2	24254.5
500	7393.1 ± 10864.1	0.0	2776.2	58849.2
1000	29875.7 ± 42708.0	0.0	15721.1	285796.6
Semi−natural & forested area (TB)				
25	311.6 ± 309.9	0.0	263.1	1611.7
50	1874.1 ± 1529.2	0.0	1603.4	6604.0
100	10280.1 ± 7064.7	352.8	8410.2	28057.3
300	137542.4 ± 57729.7	16928.6	140597.2	257485.0
500	419434.7 ± 154636.5	45407.7	439786.3	701906.5
1000	1839834.6 ± 529268.2	342241.8	1923683.7	2777905.2
All road area (ARA)				
25	350.1 ± 226.8	0.0	324.2	1143.4
50	1257.8 ± 640.8	0.0	1146.2	3038.9
100	4728.3 ± 2126.0	380.0	4535.1	10351.2
300	34223.5 ± 15345.2	8132.5	31149.6	66316.3
500	88921.3 ± 40309.5	23585.6	81220.1	183846.0
1000	311075.5 ± 121871.4	105170.3	269487.9	637921.0
Major road area (MRA)				
25	15.4 ± 102.0	0.0	0.0	789.2
50	71.7 ± 355.1	0.0	0.0	2234.4
100	281.6 ± 977.5	0.0	0.0	5018.5
300	2630.8 ± 5332.5	0.0	0.0	17539.3
500	7782.1 ± 12971.1	0.0	0.0	55389.7
1000	28103.7 ± 36430.9	0.0	8366.1	172112.1
Length of all road (AR)				
25	50.4 ± 27.1	0.0	48.0	126.0
50	182.1 ± 71.3	19.0	177.0	369.0
100	673.8 ± 227.6	122.0	672.0	1226.0
300	4706.9 ± 1401.4	1136.0	4533.0	7990.0
500	12048.6 ± 3602.6	4104.0	11410.0	20227.0
1000	44097.9 ± 11408.2	17809.0	41652.0	74886.0
Length of major road (MR)				
25	22.0 ± 26.2	0.0	0.0	94.0
50	81.7 ± 74.1	0.0	90.0	327.0
100	303.3 ± 221.1	0.0	268.0	1031.0
300	2107.6 ± 1441.4	0.0	1840.0	5764.0
500	5191.9 ± 3396.8	793.0	4444.0	14936.0
1000	17498.4 ± 9701.4	4670.0	16036.0	41782.0
Distance to the nearest all road (Distance_AR)	12.7 ± 8.2	1.4	11.0	44.4
Distance to the nearest main road (Distance_MR)	56.0 ± 68.4	3.4	27.0	305.7

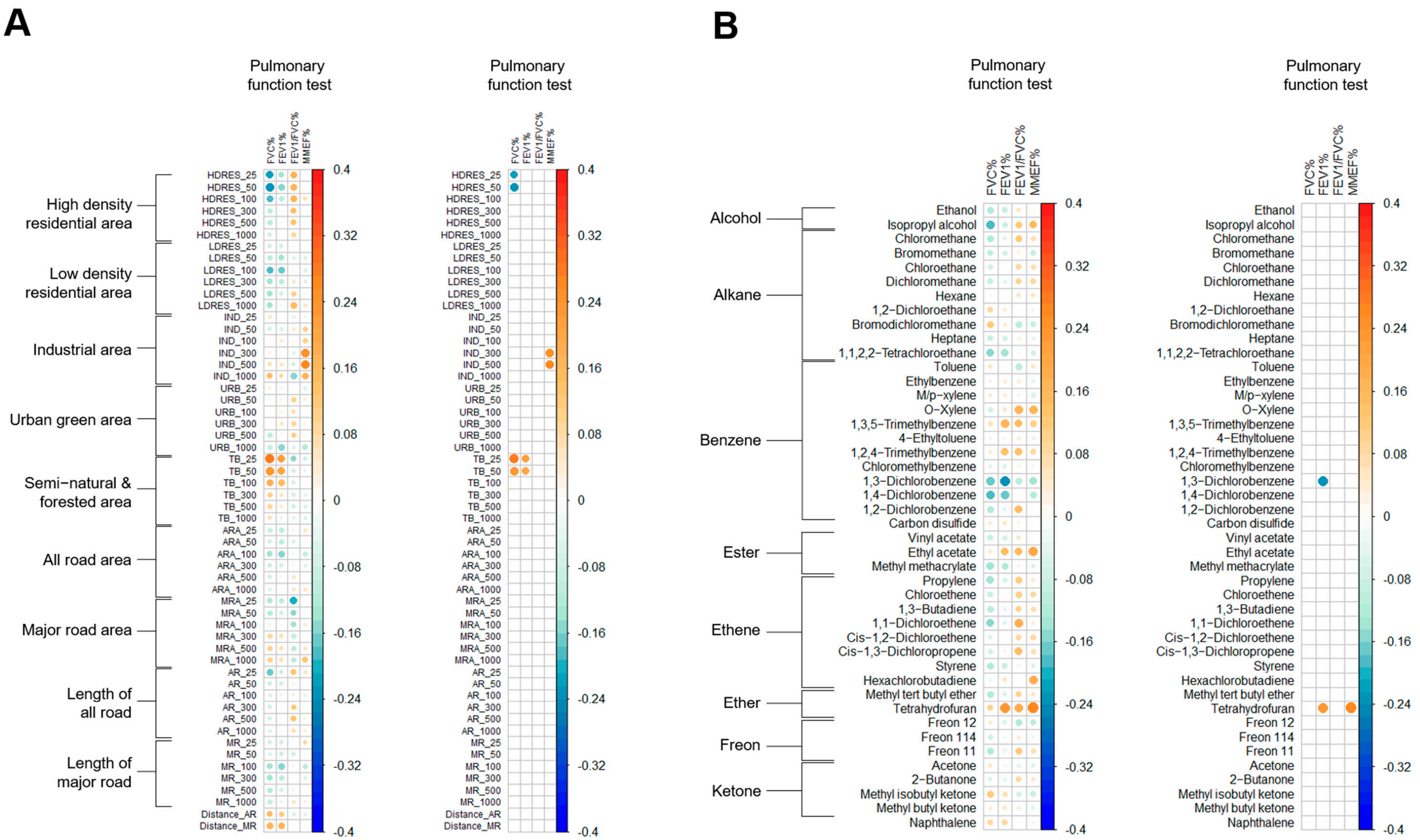

A
Pulmonary function test
FVC%
FEV1%
FEV1/FVC%
MMEF%
High density residential area
Low density residential area
Industrial area
Urban green area
Semi−natural & forested area
All road area
Major road area
Length of all road
Length of major road
HDRES_25
HDRES_50
HDRES_100
HDRES_300
HDRES_500
HDRES_1000
LDRES_25
LDRES_50
LDRES_100
LDRES_300
LDRES_500
LDRES_1000
IND_25
IND_50
IND_100
IND_300
IND_500
IND_1000
URB_25
URB_50
URB_100
URB_300
URB_500
URB_1000
TB_25
TB_50
TB_100
TB_300
TB_500
TB_1000
ARA_25
ARA_50
ARA_100
ARA_300
ARA_500
ARA_1000
MRA_25
MRA_50
MRA_100
MRA_300
MRA_500
MRA_1000
AR_25
AR_50
AR_100
AR_300
AR_500
AR_1000
MR_25
MR_50
MR_100
MR_300
MR_500
MR_1000
Distance_AR
Distance_MR
0.4
0.32
0.24
0.16
0.08
0
-0.08
-0.16
-0.24
-0.32
-0.4
B
Pulmonary function test
Alcohol
Alkane
Benzene
Ester
Ethene
Ether
Freon
Ketone
Ethanol
Isopropyl alcohol
Chloromethane
Bromomethane
Chloroethane
Dichloromethane
Hexane
1,2−Dichloroethane
Bromodichloromethane
Heptane
1,1,2,2−Tetrachloroethane
Toluene
Ethylbenzene
M/p−xylene
O−Xylene
1,3,5−Trimethylbenzene
4−Ethyltoluene
1,2,4−Trimethylbenzene
Chloromethylbenzene
1,3−Dichlorobenzene
1,4−Dichlorobenzene
1,2−Dichlorobenzene
Carbon disulfide
Vinyl acetate
Ethyl acetate
Methyl methacrylate
Propylene
Chloroethene
1,3−Butadiene
1,1−Dichloroethene
Cis−1,2−Dichloroethene
Cis−1,3−Dichloropropene
Styrene
Hexachlorobutadiene
Methyl tert butyl ether
Tetrahydrofuran
Freon 12
Freon 114
Freon 11
Acetone
2−Butanone
Methyl isobutyl ketone
Methyl butyl ketone
Naphthalene

Supplementary Figure S3. Heatmap Depicting the Correlation Coefficients between Pulmonary Function Test and (A) Land Use Variables, and (B) Exhaled Volatile Organic Compounds of 97 Children, Controlling for Age, Sex, Body Mass Index, Passive Smoking Exposure, and Insecticide Use in the Home. (The Heatmap on the Left of (A) and (B) is Presented Overall Correlation Coefficients; the Heatmap on the Right of (A) and (B) is Presented Those with Statistical Significant, $p<0.05$)

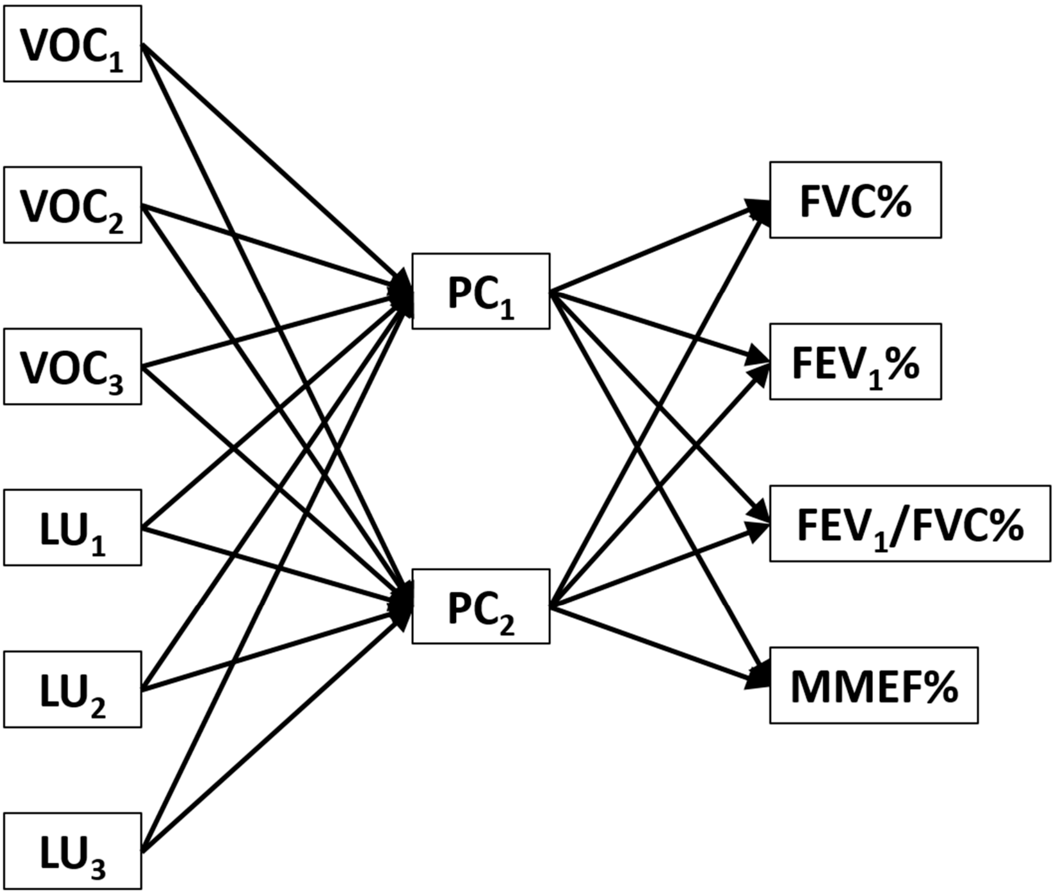

Supplementary Figure S4. Model 1 Assuming the Relationship between the Land Use Variables (LU), the Exhaled Volatile Organic Compounds (VOCs), and the Pulmonary Function Test. (PC, principal components)

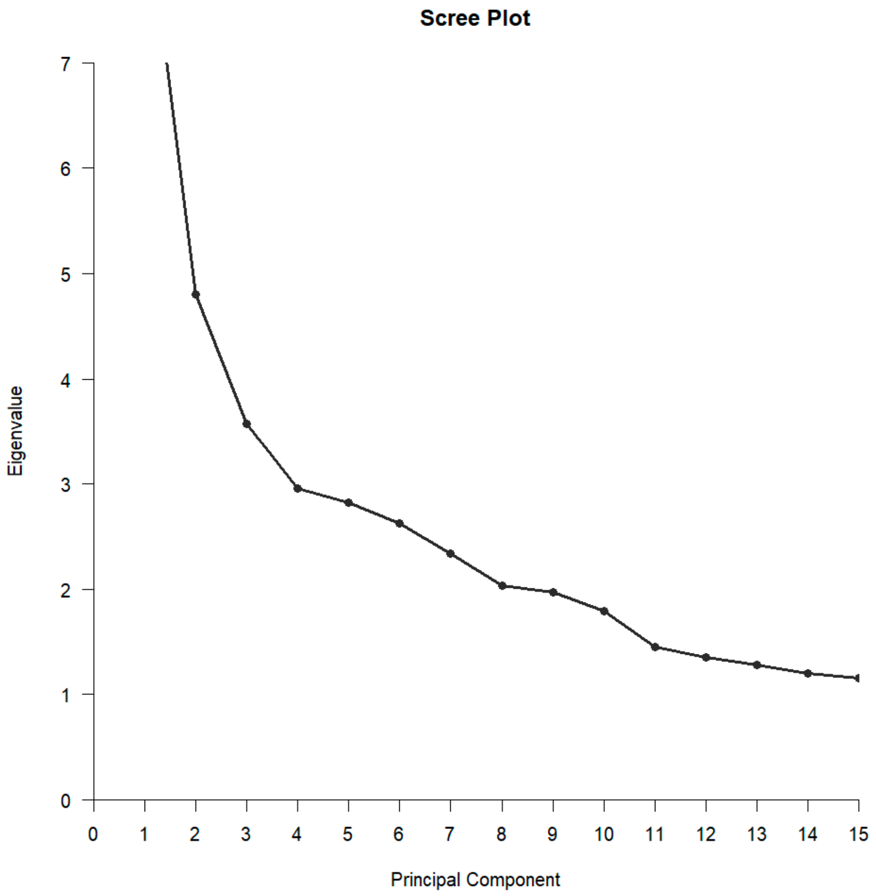

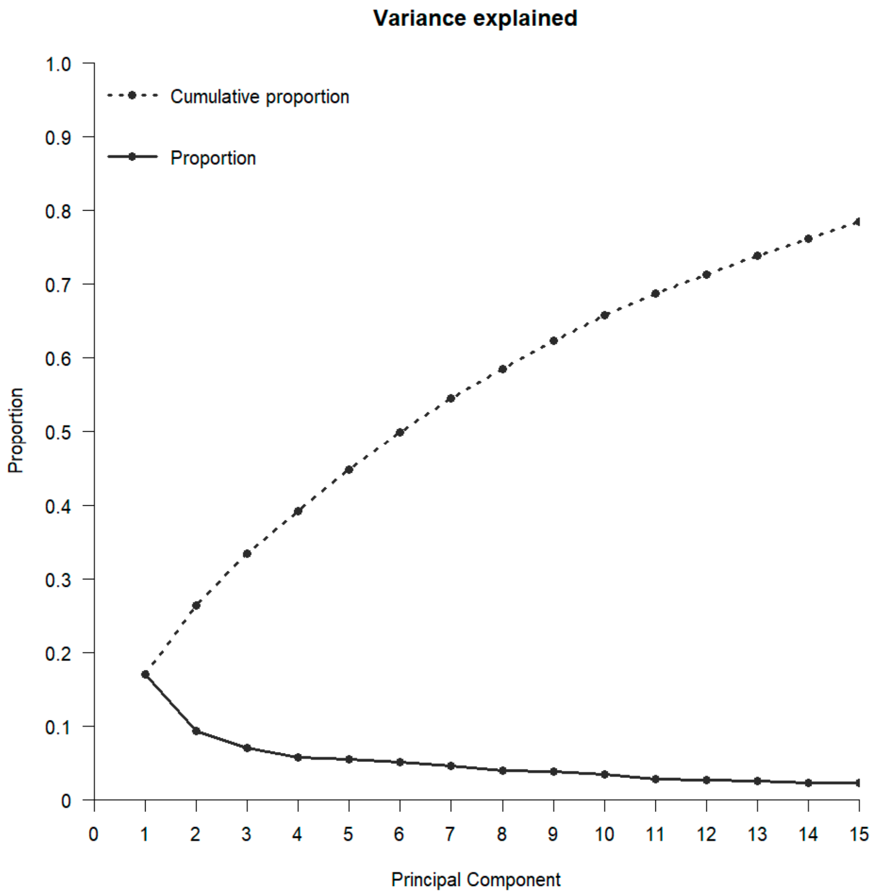

Supplementary Figure S5. The Scree Plot and the Variance Explained Plot from the Result of Principal Components Analysis from Model 1.

Supplementary Table S3. The Loading Table of 15 Principal Components from Model 1.

**Variables**	**PC_1**	**PC_2**	**PC_3**	**PC_4**	**PC_5**	**PC_6**	**PC_7**	**PC_8**	**PC_9**	**PC_{10}**	**PC_{11}**	**PC_{12}**	**PC_{13}**	**PC_{14}**	**PC_{15}**
Isopropyl alcohol	0.096	-0.020	0.058	0.203	-0.037	0.171	-0.060	0.091	-0.037	-0.144	-0.349	-0.004	0.225	-0.123	0.152
Bromomethane	-0.029	-0.179	0.121	0.119	0.035	0.278	-0.227	0.005	-0.282	-0.138	0.203	-0.106	-0.018	-0.045	0.016
Hexane	-0.027	-0.074	-0.007	0.029	-0.052	0.065	0.247	0.141	-0.325	0.001	0.117	-0.341	0.078	-0.009	0.294
1,2−Dichloroethane	0.047	0.011	0.064	0.073	-0.158	-0.319	0.282	0.076	-0.239	0.045	-0.164	0.119	0.050	-0.057	-0.153
Bromodichloromethane	0.026	0.028	-0.061	-0.076	-0.008	0.033	-0.087	-0.278	0.029	0.031	-0.081	-0.129	0.030	0.380	0.395
M/p−xylene	0.022	0.076	0.250	0.020	0.275	-0.090	0.148	-0.119	-0.062	0.047	0.145	0.331	0.021	-0.043	0.234
1,3,5−Trimethylbenzene	0.073	0.090	0.284	-0.028	0.358	-0.088	0.123	0.022	0.086	-0.071	0.149	0.040	0.049	-0.070	0.186
4−Ethyltoluene	0.011	0.047	0.098	0.091	0.321	-0.036	0.102	0.077	0.177	-0.165	0.099	-0.297	0.089	0.038	-0.266
1,2,4−Trimethylbenzene	0.034	0.087	0.260	0.016	0.364	-0.133	0.162	0.099	0.133	-0.065	0.010	-0.023	-0.060	-0.048	0.111
Chloromethylbenzene	0.028	-0.080	0.047	0.100	0.063	0.039	0.123	-0.190	0.161	-0.036	-0.399	0.003	0.349	-0.114	-0.173
1,4−Dichlorobenzene	-0.030	-0.122	-0.049	-0.177	-0.062	0.099	0.007	0.028	0.227	0.152	0.281	0.084	0.068	-0.003	0.253
1,2−Dichlorobenzene	-0.004	-0.049	-0.131	0.022	0.151	0.177	-0.103	0.139	0.103	0.465	0.155	0.034	0.065	-0.076	0.049
Carbon disulfide	0.028	-0.121	0.335	0.189	0.059	0.146	-0.122	-0.131	-0.172	0.069	0.001	0.165	-0.008	0.048	-0.043
Vinyl acetate	0.007	-0.044	0.243	0.327	-0.006	-0.153	-0.123	0.230	0.104	0.047	-0.064	-0.080	-0.167	0.207	-0.006
Methyl methacrylate	0.065	0.119	-0.010	0.037	-0.076	0.116	0.109	0.237	0.066	-0.179	-0.053	0.322	-0.286	0.264	-0.066
1,1−Dichloroethene	-0.018	-0.041	0.025	0.221	0.189	0.167	-0.172	0.181	0.055	0.347	-0.108	-0.073	0.095	0.039	0.131
Tetrahydrofuran	0.007	-0.026	-0.050	0.034	-0.035	-0.017	0.301	0.190	-0.330	0.023	-0.055	-0.144	0.318	0.148	0.137
Freon 12	-0.022	-0.024	-0.070	-0.002	0.102	0.017	-0.153	-0.050	0.093	-0.290	0.298	-0.024	0.481	0.165	-0.184
Freon 114	-0.015	-0.171	0.182	0.151	0.016	0.274	-0.238	-0.018	-0.228	-0.088	0.033	0.031	-0.029	0.058	-0.109
2−Butanone	0.011	-0.026	0.094	0.251	-0.091	-0.266	-0.196	0.218	0.149	0.111	-0.167	-0.133	-0.020	0.093	0.119
Methyl isobutyl ketone	0.029	0.011	0.316	-0.032	-0.110	-0.019	-0.083	-0.079	-0.247	0.079	0.044	0.328	0.099	-0.134	-0.076
HDRES_25	-0.054	-0.322	0.146	-0.034	-0.117	0.033	0.185	0.027	0.153	-0.080	0.074	0.041	0.063	-0.009	-0.066
HDRES_50	0.048	-0.354	0.102	0.036	-0.130	0.058	0.212	-0.027	0.126	-0.051	0.057	0.018	-0.056	0.110	0.016
HDRES_100	0.123	-0.320	0.045	0.089	-0.077	0.012	0.124	-0.132	0.185	-0.018	-0.041	0.022	-0.034	0.117	0.104
HDRES_300	0.247	-0.178	0.047	-0.056	0.069	-0.019	0.009	-0.032	0.100	-0.039	-0.092	0.058	-0.035	0.017	0.039
LDRES_500	0.257	-0.002	-0.011	-0.186	0.098	-0.046	-0.129	0.150	-0.118	-0.081	-0.032	-0.128	-0.076	-0.124	-0.048
LDRES_1000	0.258	0.004	0.045	-0.189	0.069	-0.083	-0.107	0.152	-0.105	-0.088	-0.012	-0.155	-0.097	-0.089	-0.001
IND_25	-0.024	0.054	-0.044	0.154	-0.167	-0.336	-0.195	0.084	-0.029	-0.059	0.252	0.111	0.189	-0.084	0.030
IND_50	-0.028	0.069	-0.069	0.242	-0.198	-0.299	-0.172	0.055	0.037	-0.089	0.250	0.117	0.153	-0.058	0.150
URB_25	0.015	0.008	-0.128	0.117	0.123	0.032	-0.004	0.007	0.056	0.370	-0.002	0.015	0.028	-0.250	-0.291
URB_300	0.115	-0.136	-0.074	-0.090	-0.015	0.025	0.103	0.247	-0.034	0.133	-0.092	0.194	0.332	-0.015	0.037
URB_500	0.130	-0.118	-0.122	-0.140	0.075	0.046	0.039	0.347	0.028	0.058	0.031	0.312	0.081	0.212	0.014
URB_1000	0.157	-0.080	-0.067	-0.170	0.085	0.052	-0.152	0.248	-0.016	-0.153	-0.012	0.069	0.046	0.187	-0.146
TB_25	-0.169	0.192	-0.020	-0.113	0.099	0.065	-0.138	-0.016	-0.033	-0.143	-0.204	0.145	0.054	0.077	0.200
TB_50	-0.252	0.158	-0.012	-0.156	0.155	0.063	-0.075	0.045	-0.048	-0.038	-0.137	0.114	0.088	0.046	0.032
TB_100	-0.280	0.106	0.034	-0.097	0.090	0.066	0.031	0.102	-0.094	0.000	-0.062	0.044	0.093	0.087	-0.125
TB_300	-0.313	0.048	0.035	0.082	-0.023	0.040	0.088	-0.024	0.049	0.001	0.021	0.014	0.022	0.089	-0.021
TB_500	-0.305	0.004	0.029	0.113	-0.013	0.037	0.131	-0.056	0.103	0.001	0.007	0.010	0.065	0.023	-0.003
ARA_25	0.162	0.200	-0.161	0.214	0.078	0.059	0.128	-0.112	-0.141	0.063	0.052	0.011	-0.074	0.140	0.075
ARA_50	0.235	0.158	-0.067	0.140	0.008	0.075	0.034	-0.139	-0.091	-0.020	-0.048	0.009	0.074	-0.056	0.087
ARA_100	0.298	0.043	-0.051	0.015	-0.005	0.032	-0.065	-0.065	-0.025	-0.033	0.001	0.055	0.020	-0.124	0.072
MRA_25	0.040	0.230	0.062	0.104	-0.238	0.287	0.093	0.108	0.145	-0.071	0.058	0.108	-0.010	-0.011	0.016
MRA_50	0.074	0.239	0.092	0.058	-0.230	0.303	0.063	0.121	0.173	-0.095	0.014	-0.017	0.058	-0.151	0.036
MRA_100	0.088	0.258	0.160	0.004	-0.163	0.186	0.044	0.184	0.203	-0.059	0.062	-0.161	0.047	-0.147	0.027
MRA_300	0.112	0.210	0.272	-0.131	-0.132	-0.004	0.020	0.000	0.057	0.122	0.068	-0.058	0.135	0.022	0.015
MRA_500	0.114	0.151	0.251	-0.222	-0.170	-0.049	-0.043	-0.074	-0.012	0.233	-0.016	-0.101	0.115	0.244	-0.113
MRA_1000	0.085	0.091	0.241	-0.193	-0.139	-0.041	-0.099	-0.140	0.007	0.231	-0.003	-0.127	0.110	0.310	-0.164
AR_100	0.237	-0.009	-0.102	-0.011	-0.005	0.020	-0.043	-0.283	0.128	-0.021	-0.012	0.081	0.083	-0.135	0.064
MR_25	0.119	0.189	-0.135	0.220	0.054	0.070	0.197	-0.053	-0.131	0.114	0.139	0.077	-0.121	0.187	-0.194
Distance_AR	-0.131	-0.098	0.139	-0.202	-0.107	0.068	0.137	0.063	-0.091	0.090	0.137	-0.092	-0.122	-0.249	0.006
Distance_MR	-0.184	-0.032	0.084	-0.159	-0.080	-0.071	-0.163	0.086	0.023	-0.007	-0.240	0.054	-0.083	-0.173	0.155

Abbreviation: PC, principal component

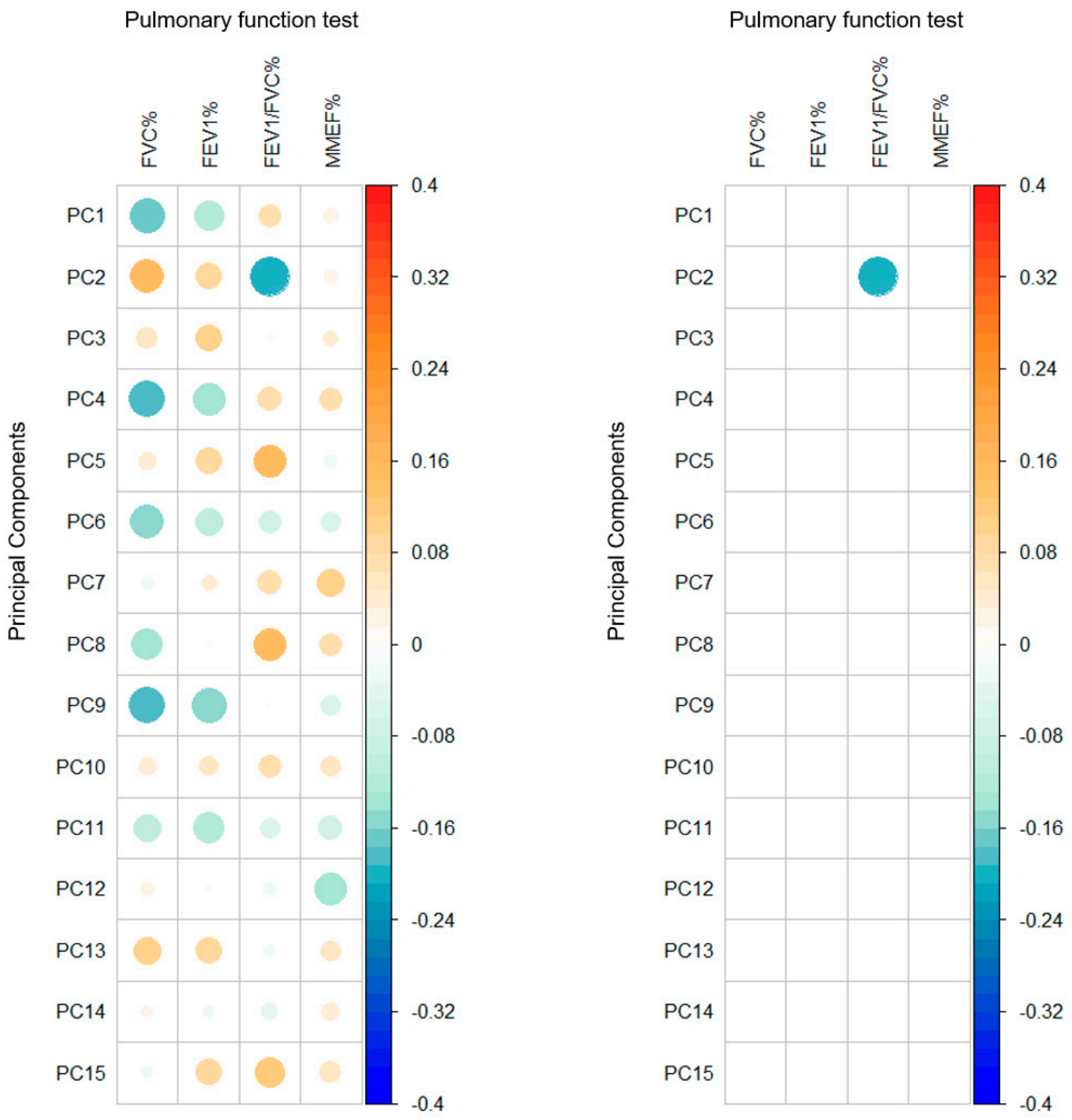

Supplementary Figure S6. Heatmap Depicting the Correlation Coefficients between 15 Principal Components and the Pulmonary Function Test. (A) Overall Correlation Coefficients; (B) Those with Statistical Significant, p<0.05

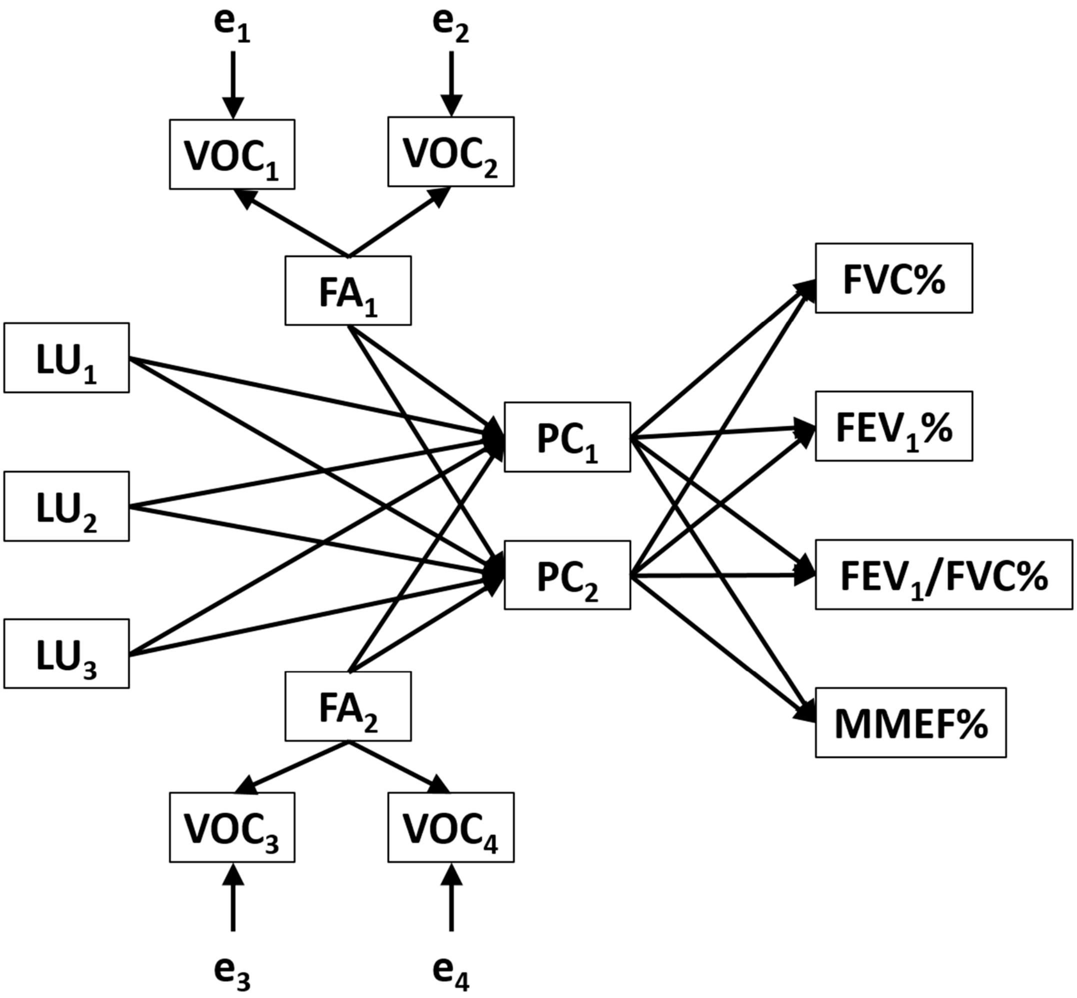

Supplementary Figure S7. Model 2 Assuming the Relationship between the Land Use Variables (LU), the Precursors (Factors) of the Exhaled Volatile Organic Compounds (VOCs), and the Pulmonary Function Test. (FA, Factor; PC, principal component; e, measurement errors)

Supplementary Table S4. The Loading Table of 10 Principal Components Including 5 Factors from Model 2.

**Variables**	**PC_1**	**PC_2**	**PC_3**	**PC_4**	**PC_5**	**PC_6**	**PC_7**	**PC_8**	**PC_9**	**PC_{10}**
Factor 1	0.042	0.067	0.105	-0.098	0.059	-0.236	0.081	0.227	0.523	0.064
Factor 2	0.002	-0.113	0.102	0.092	0.071	-0.166	-0.115	-0.070	0.234	0.063
Factor 3	0.005	-0.017	-0.033	0.211	-0.231	0.167	0.068	0.404	0.347	0.235
Factor 4	-0.037	-0.055	-0.159	-0.140	0.244	0.137	-0.147	0.383	0.145	-0.106
Factor 5	0.006	-0.053	-0.066	0.038	0.158	0.019	0.548	-0.279	0.213	-0.097
HDRES_25	-0.052	-0.339	0.201	0.208	0.091	0.062	0.007	-0.068	0.050	-0.073
HDRES_50	0.053	-0.367	0.129	0.272	0.126	-0.009	-0.009	-0.053	0.020	0.102
HDRES_100	0.129	-0.325	0.033	0.245	0.056	-0.084	-0.117	0.072	-0.082	0.226
HDRES_300	0.252	-0.186	0.062	-0.031	0.019	-0.014	-0.112	0.111	-0.002	0.114
LDRES_500	0.262	-0.002	0.069	-0.279	-0.059	0.069	-0.003	-0.112	0.192	-0.129
LDRES_1000	0.261	0.002	0.122	-0.236	-0.101	0.044	0.033	-0.109	0.219	-0.116
IND_25	-0.024	0.056	-0.114	0.129	-0.516	0.219	0.118	-0.050	0.010	-0.093
IND_50	-0.030	0.077	-0.171	0.231	-0.468	0.221	0.038	-0.061	-0.003	0.006
URB_25	0.017	0.010	-0.205	-0.016	0.091	0.024	0.086	0.564	-0.173	-0.470
URB_300	0.120	-0.138	-0.001	-0.089	0.112	0.337	0.304	0.065	-0.272	0.137
URB_500	0.135	-0.139	-0.034	-0.200	0.114	0.398	0.202	0.087	-0.126	0.213
URB_1000	0.162	-0.085	0.043	-0.270	0.022	0.249	-0.081	-0.008	0.070	0.227
TB_25	-0.175	0.188	0.008	-0.238	0.012	0.021	-0.203	-0.095	-0.096	0.277
TB_50	-0.257	0.144	0.023	-0.283	0.073	0.026	-0.052	-0.010	-0.043	0.115
TB_100	-0.286	0.091	0.057	-0.161	0.103	0.039	0.127	-0.013	0.027	0.038
TB_300	-0.319	0.041	-0.008	0.112	0.069	-0.025	0.025	0.023	0.004	0.054
TB_500	-0.310	-0.005	-0.029	0.157	0.089	-0.025	-0.002	0.054	-0.003	-0.006
ARA_25	0.160	0.217	-0.296	0.083	0.130	-0.169	0.133	-0.074	0.029	0.180
ARA_50	0.233	0.181	-0.143	0.077	0.068	-0.102	-0.003	-0.148	-0.014	-0.062
ARA_100	0.301	0.062	-0.058	-0.012	-0.007	-0.002	-0.113	-0.098	-0.031	-0.144
MRA_25	0.034	0.264	0.094	0.279	0.240	0.288	-0.124	-0.056	-0.045	0.106
MRA_50	0.068	0.277	0.159	0.242	0.238	0.310	-0.169	-0.063	0.026	-0.054
MRA_100	0.082	0.293	0.228	0.169	0.137	0.258	-0.080	0.057	0.192	-0.117
MRA_300	0.106	0.232	0.348	0.067	-0.047	-0.043	0.132	0.091	0.042	-0.080
MRA_500	0.111	0.169	0.393	-0.003	-0.122	-0.159	0.211	0.171	-0.241	0.110
MRA_1000	0.084	0.113	0.359	-0.002	-0.140	-0.229	0.120	0.225	-0.266	0.171
AR_100	0.242	0.002	-0.082	0.033	0.009	-0.119	-0.313	-0.025	-0.253	-0.181
MR_25	0.118	0.204	-0.270	0.125	0.187	-0.152	0.238	0.027	-0.077	0.234
Distance_AR	-0.129	-0.107	0.251	-0.002	0.128	0.034	0.231	-0.136	-0.107	-0.381
Distance_MR	-0.186	-0.044	0.172	-0.125	-0.146	0.120	-0.193	-0.053	0.002	-0.029

Abbreviation: PC, principal component

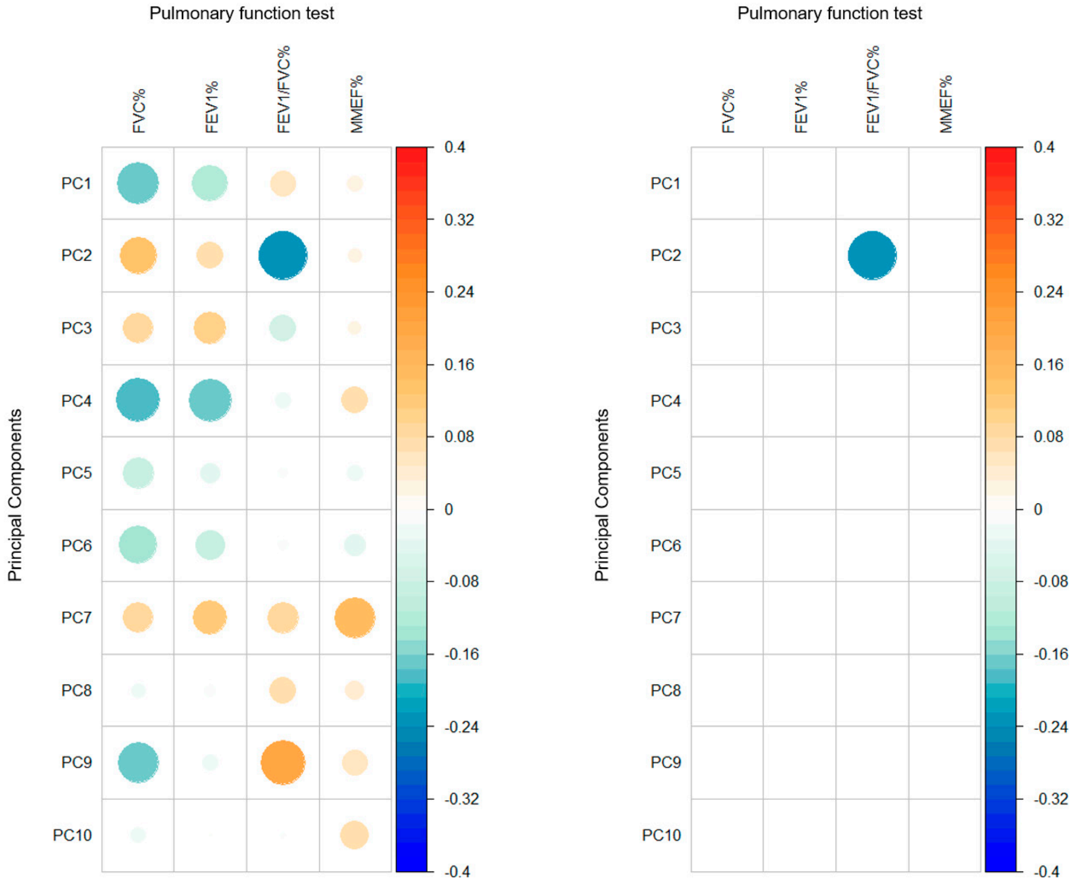

Supplementary Figure S8. Heatmap Depicting the Correlation Coefficients between the 10 Principal Components and the Pulmonary Function Test. (A) Overall Correlation Coefficients; (B) Those with Statistical Significant, $p<0.05$

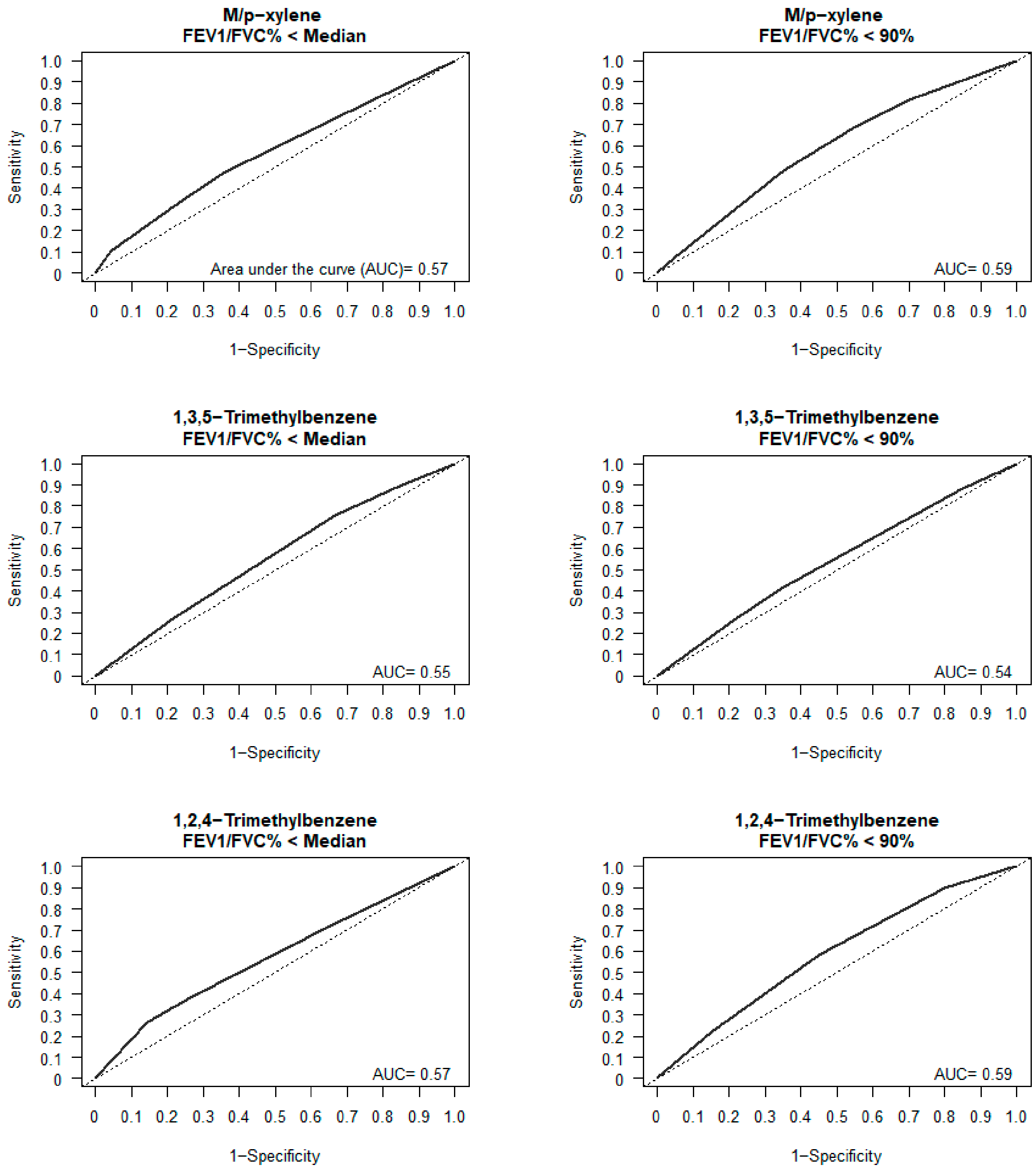

Supplementary Figure S9. Receiver Operating Characteristic Curves (ROC) for the Abnormal FEV_1/FVC% Criteria (<Median (83.7%) or <90%) Based on M/p−Xylene (Categories: ≤ 0.01, 0.02−0.03, 0.04−0.07, 0.08−0.16, 0.17+ ppb), 1,3,5−Trimethylbenzene (Categories: ≤ 0.01, 0.02−0.03, 0.04−0.05, 0.06+ ppb), and 1,2,4−Trimethylbenzene

(Categories: ≤0.01, 0.02−0.03, 0.04−0.08, 0.09+ ppb), respectively.

Supplementary Table S5. Predicted Model for Abnormal FEV_1/FVC% (<Median or <90%) Based on M/p−Xylene, 1,3,5−Trimethylbenzene, and 1,2,4−Trimethylbenzene, respectively.

VOCs	Number of children (N=97) (%)	FEV_1/FVC% < Median (83.7%)			FEV_1/FVC% < 90%		
		Coefficient	p value	AUC (95% Wald CI)	Coefficient	p value	AUC (95% Wald CI)
M/p−xylene, ppb							
≤ 0.01	20 (20.6)	Reference		0.57 (0.46−0.68)	Reference		0.59 (0.45−0.73)
0.02－0.03	17 (17.5)	1.12	0.24		0.41	0.74	
0.04－0.07	27 (27.8)	0.04	0.95		0.26	0.72	
0.08－0.16	15 (15.5)	0.35	0.62		−0.18	0.83	
0.17+	18 (18.6)	0.40	0.53		−0.54	0.47	
Intercept		−0.20	0.66		1.39	0.01	
1,3,5−trimethylbenzene, ppb							
≤ 0.01	46 (47.4)	Reference		0.55 (0.45−0.66)	Reference		0.54 (0.41−0.67)
0.02－0.03	23 (23.7)	0.18	0.73		0.28	0.67	
0.04－0.05	12 (12.4)	−0.42	0.52		−0.18	0.81	
0.05+	16 (16.5)	−0.34	0.56		0.19	0.80	
Intercept		0.09	0.77		1.28	0.0003	
1,2,4−trimethylbenzene, ppb							
≤ 0.01	40 (41.2)	Reference		0.57 (0.46−0.68)	Reference		0.59 (0.45−0.73)
0.02－0.03	25 (25.8)	0.08	0.88		0.31	0.62	
0.04－0.08	13 (13.4)	0.19	0.78		−0.54	0.47	
0.09+	19 (19.6)	0.81	0.17		0.50	0.51	
Intercept		−0.19	0.59		1.23	0.004	

Abbreviation: VOCs, volatile organic compounds; FEV_1, forced expiratory volume in one second; FVC, forced vital capacity; AUC, area under the receiver operating characteristic curves

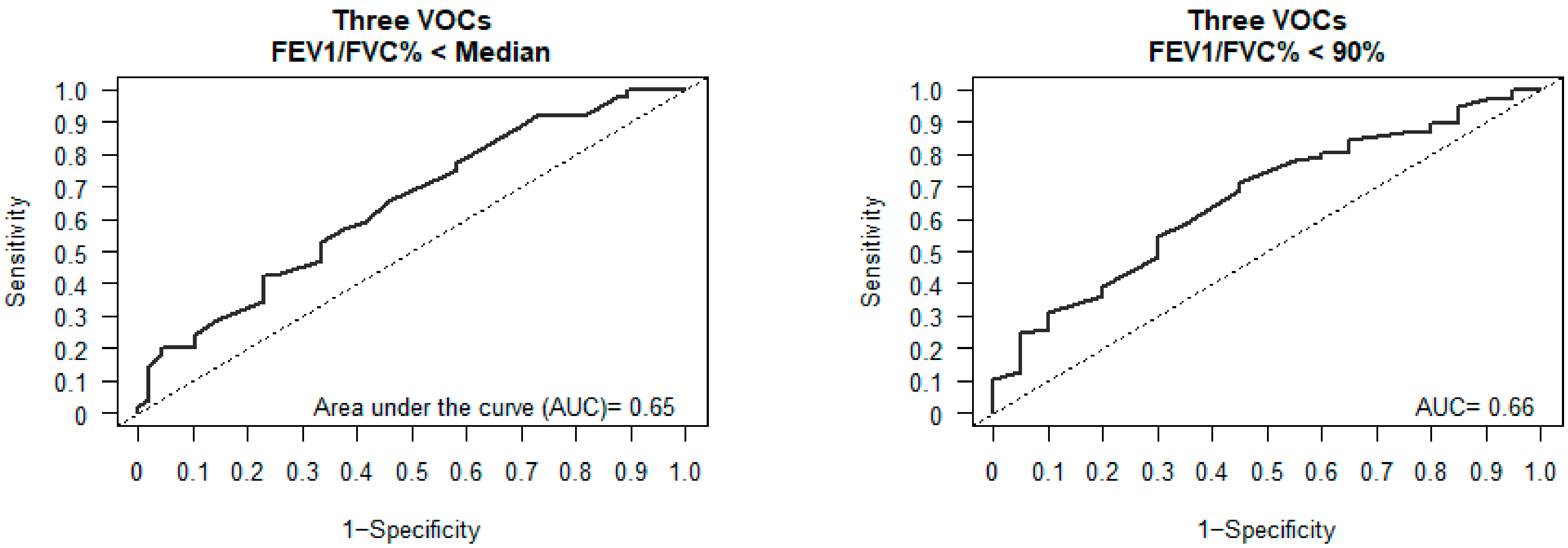

Supplementary Figure S10. Receiver Operating Characteristic (ROC) Curves for the Abnormal FEV$_1$/FVC% Criteria (<Median (83.7%) or <90%) Considering Simultaneously M/p-Xylene (Categories: ≤ 0.01, 0.02−0.03, 0.04−0.07, 0.08−0.16, 0.17+ ppb), 1,3,5-Trimethylbenzene (Categories: ≤ 0.01, 0.02−0.03, 0.04−0.05, 0.06+ ppb), and 1,2,4-Trimethylbenzene (Categories: ≤0.01, 0.02−0.03, 0.04−0.08, 0.09+ ppb).

Supplementary Table S6. Predicted Model for Abnormal FEV_1/FVC% (<Median or <90%) Considering Simultaneously M/p−Xylene, 1,3,5−Trimethylbenzene, and 1,2,4−Trimethylbenzene.

VOCs	FEV_1/FVC% < Median (83.7%)			FEV_1/FVC% < 90%		
	Coefficient	p value	AUC (95% Wald CI)	Coefficient	p value	AUC (95% Wald CI)
Intercept	−0.20	0.70	0.65 (0.54−0.75)	1.41	0.01	0.66 (0.53−0.79)
M/p−xylene, ppb						
0.02 − 0.03 vs. ≤ 0.01	1.19	0.24		0.22	0.41	
0.04 − 0.07 vs. ≤ 0.01	0.05	0.93		0.17	0.95	
0.08 − 0.16 vs. ≤ 0.01	−0.13	0.87		−0.49	0.83	
0.17+ vs. ≤ 0.01	0.13	0.87		−1.05	0.16	
1,3,5−trimethylbenzene, ppb						
0.02 − 0.03 vs. ≤ 0.01	−0.09	0.88		0.28	0.92	
0.04 − 0.05 vs. ≤ 0.01	−0.78	0.30		−0.11	0.83	
0.05+ vs. ≤ 0.01	−1.38	0.10		−0.02	0.84	
1,2,4−trimethylbenzene, ppb						
0.02 − 0.03 vs. ≤ 0.01	0.14	0.81		0.05	0.32	
0.04 − 0.08 vs. ≤ 0.01	0.51	0.49		−0.49	0.35	
0.09+ vs. ≤ 0.01	1.68	0.05		1.05	0.43	

Abbreviation: VOCs, volatile organic compounds; FEV_1, forced expiratory volume in one second; FVC, forced vital capacity; AUC, area under the receiver operating characteristic curves
